# Microglial SWELL1 deficiency drives male-specific seizure vulnerability but paradoxical neuroprotection through impaired phagocytosis

**DOI:** 10.1172/jci.insight.197980

**Published:** 2026-06-22

**Authors:** Abhijeet S. Barath, Aastha Dheer, Laura Montier, Mekenzie M Peshoff, Emily Dale, Flavia Goche, Thanh Thanh Le Nguyen, Mastura Akter, FangFang Qi, Dimitrios Kleidonas, Lauren Harris, Sarah A. Jewanee, Rongzhuo Hua, Tejaswani Datla, Anthony D. Umpierre, Dale B. Bosco, Koichiro Haruwaka, Rajan Sah, Long-Jun Wu

**Affiliations:** 1 Department of Neurology, Mayo Clinic, Rochester, Minnesota, USA.; 2 Mayo Clinic Graduate School of Biomedical Sciences, Rochester, Minnesota, USA.; 3 Center for Neuroimmunology and Glial Biology, Institute of Molecular Medicine, University of Texas Health Science Center at Houston, Houston, Texas, USA.; 4 Department of Neurology, Yale New Haven Hospital, New Haven, Connecticut, USA.; 5 MD Anderson UTHealth Graduate School of Biomedical Sciences, Houston, Texas, USA.; 6 Department of Genetics, Yale School of Medicine, New Haven, Connecticut, USA.; 7 Department of Internal Medicine, Cardiovascular Division, Washington University School of Medicine, St. Louis, Missouri, USA.

**Keywords:** Immunology, Neuroscience, Epilepsy, Seizures

## Abstract

The discovery of genes encoding the volume-regulated anion channel (VRAC) has enabled detailed exploration of its cell type–specific roles in the brain. LRRC8A (SWELL1) is the essential VRAC subunit. We observed seizure-induced, subunit-specific changes in microglial VRAC expression and investigated its function using conditional KO (cKO) of LRRC8A in microglia. SWELL1 cKO mice exhibited a male-specific increase in kainate-induced seizure severity, yet showed paradoxical neuroprotection against seizure-associated neuronal loss. Mechanistically, SWELL1 deletion led to a cell-autonomous reduction in microglial density and decreased release of VRAC-permeable neuroactive metabolites, including taurine, GABA, and glutamate in culture. Additionally, impaired phagocytic kinetics and reduced lysosomal biogenesis contributed to the observed neuroprotection. These findings reveal potentially novel roles for microglial VRAC in regulating seizure outcomes and microglia-neuron interactions.

## Abstract 2

A swell-activated anion channel (SWELL1) in microglia, a resident brain immune cell, reduces seizure severity but increases neuronal loss in male mice.

## Graphical abstract

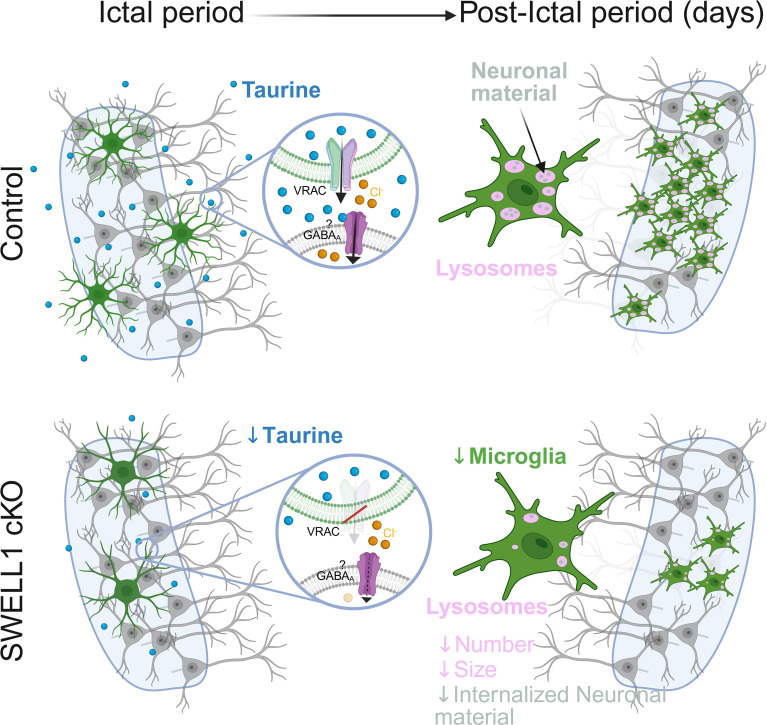

## Introduction

Microglia, as the primary immune cells of the CNS, have long been studied for their role in neuroinflammation and neurodegeneration after seizures (1–3). The emergence of seizures as a clinical manifestation in primary microgliopathies such as Nasu-Hakola disease (TREM2/DAP12 or triggering receptor expressed on myeloid cells 2/DNAX activating protein of 12 kDa dysfunction) and adult-onset leukoencephalopathy with axonal spheroids and pigmented glia (associated with CSF1R haploinsufficiency) has further underscored their relevance in acute seizure pathophysiology (4, 5). Interest in this area has intensified, with findings showing that microglial ablation exacerbates kainite-induced (KA-induced) seizures in adult mice (6–8). Several microglial signaling pathways, including P2Y12R/ATP, CX3CR1/CX3CL1, TREM2, and Gi-coupled GPCRs, have been implicated in modulating seizure severity (1, 2, 7, 9–14). Notably, recent clinical data has shown that P2Y12 receptor polymorphisms influence seizure phenotype in patients, supporting these preclinical findings (15).

After a seizure, microglia contribute to tissue remodeling by clearing damaged neurons via phagocytic receptors and releasing inflammatory cytokines (3, 11, 16). Microglial TREM2 and P2Y6, as well as Gi-coupled GPCR signaling, have been directly implicated in regulating neuronal loss after seizures (11, 12, 16). Whether microglial phagocytosis is beneficial or detrimental remains debated and likely depends on the outcome measured. For instance, P2Y6 deficiency impaired microglial phagocytosis, resulting in reduced neuronal loss, which was associated with improved hippocampal-dependent memory in the weeks after status epilepticus (16). However, TREM2 loss, which was also associated with impaired phagocytosis and neuroprotection, led to an increased risk of spontaneous recurrent seizures (11). Similarly, microglia play a role in promoting aberrant neurogenesis after seizures, which increases susceptibility to spontaneous recurrent seizures (2, 3, 11, 17–22).

The volume-regulated anion channel (VRAC) is a heterogenous, multi-subunit channel, which classically mediates regulatory volume decrease of cells under conditions of hypoosmotic stress (23). It is encoded by the members of *Lrrc8* gene family (*Lrrc8a-e*) (24, 25). The SWELL1 protein, encoded by the *Lrrc8a* gene, is essential for functional VRAC channel formation (23–25). In our previous study, bulk RNA-seq after KA-induced status epilepticus revealed complex changes in the expression of all subunits of VRAC expressed in microglia (11). VRAC is broadly expressed across mammalian cell types, and recent studies have highlighted its diverse and context-dependent roles, including regulating astrocytic glutamate release, influencing stroke outcomes, and mediating mechanosensation in vascular endothelium (24–28). Pharmacological studies have further implicated VRAC in cellular migration, motility, and proliferation (29–31) — functions particularly relevant to microglial responses during seizures. These findings prompted us to investigate the role of microglial VRAC in acute seizures and subsequent pathology. Here, we employed advanced transgenic mouse models to selectively delete *Lrrc8a* in microglia. Our results demonstrate that microglial SWELL1 critically modulates both acute seizure severity and postseizure neuropathology, likely via mechanistically distinct pathways.

## Results

### Seizures downregulate microglial SWELL1 expression while microglia-specific SWELL1 deficiency leads to increased seizure severity.

**Figure 1:**
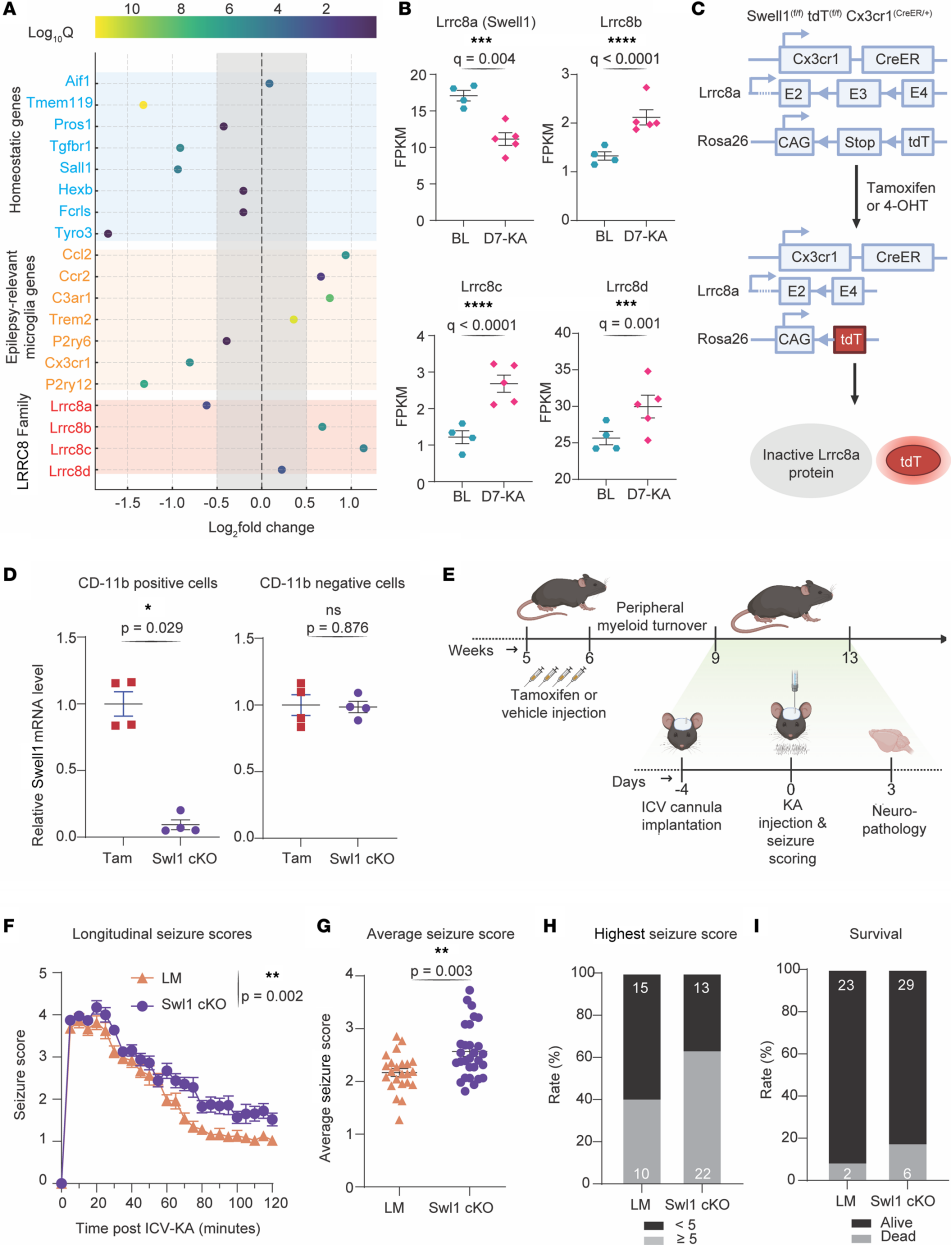
Bidirectional interplay between microglial VRAC expression and seizure severity. (**A**) A dot plot showing changes in microglial homeostatic, epilepsy-related, and *Lrrc8* family genes at day 7 after kainate-induced seizure in WT mice (bulk sequencing of brain CD11b^+^ cells; *n* = 4–5 WT mice/group). (**B**) *Lrrc8a*-*Lrrc8d* expression in microglia at day 7 after seizures (statistic: *q* value [i.e., FDR-adjusted *P* value]). (**C**) Swell1 cKO mouse stop codons are placed around the E3 exon, which is spliced out after tamoxifen or 4-OHT treatment. tdTomato expression is also activated. (**D**) Relative Swell1 mRNA levels in CD11b^+^ and CD11b^–^ cells showing cKO efficiency and selectivity (mRNA normalized to GAPDH) versus control animals treated with tamoxifen (statistic: Mann-Whitney *U* test and unpaired 2-tailed *t* test). (**E**) Seizure experiment timeline. (**F**) Longitudinal Racine seizure scores in SWELL1 cKO versus littermate controls (*n* = 25–35 mice/group; statistic: 2-way ANOVA). (**G**) Average seizure scores over the 2-hour observation period (statistic: unpaired 2-tailed *t* test). (**H**) Percentage of mice achieving a Racine score of 5 or higher at least once. (**I**) Survival rate. 4-OHT: 4-hydroxy tamoxifen (active metabolite of tamoxifen); FPKM, fragments per kilobase of exon per million mapped fragments; ICV, intracerebroventricular; KA, kainate; LM, vehicle-treated littermate controls; Tam, tamoxifen-treated controls. *P <0.05, **P <0.01, ***P or *q* <0.005, ****P or *q* <0.001

Microglia are known to regulate neuronal excitability during acute seizures and coordinate neuroimmune responses during the postictal period (1–3, 21). To explore the role of microglial VRAC in this context, we analyzed our previously published bulk RNA-seq data from CD11b^+^ cells isolated from WT mice before and 7 days after KA-induced seizures (11). This analysis revealed significant alterations in homeostatic and seizure-associated gene expression, as well as in all *Lrrc8* family members expressed in microglia (Figure 1A). At baseline, *Lrrc8a* and *Lrrc8d* exhibited the highest expression levels among the VRAC subunits, with lower levels of *Lrrc8b* and *Lrrc8c* (Figure 1B). A similar subunit expression pattern was observed at the protein level in ex vivo and primary cultured microglia using publicly available proteomic data (Supplemental Figure 1C; supplemental material available online with this article; https://doi.org/10.1172/jci.insight.197980DS1) (32). Seven days after seizure induction, *Lrrc8a*, the obligate VRAC subunit, was significantly downregulated, and *Lrrc8* subunits *b-d* were upregulated (Figure 1B), suggesting seizure-induced reconfiguration of VRAC subunit composition in microglia.

To investigate the functional consequences of this shift, we generated a tamoxifen-inducible, SWELL1 conditional KO (cKO) by crossing SWELL1^(fl/fl)^ mice with CX3CR1^(Cre-ER/Cre-ER)^ mice (Figure 1, C and E). This approach enabled selective deletion of SWELL1 in adult microglia. qPCR confirmed a robust and selective reduction in SWELL1 mRNA in CD11b^+^ brain cells after tamoxifen administration, achieving an average knockdown efficiency of 91% ± 8% (Figure 1D). Functional in vitro validation demonstrated impaired regulatory volume decrease in SWELL1-deficient microglia when exposed to a mild hypotonic challenge (osmolarity 200–215 mOsm/L; Supplemental Figure 1, A and B, and Supplemental Videos 1 and 2). SWELL1 cKO microglia displayed much slower kinetics of volume reduction and larger cell sizes than controls.

To assess the in vivo impact of microglial SWELL1 loss, we employed the intracerebroventricular KA (ICV-KA) model of temporal lobe epilepsy. This model produces a seizure for a few hours followed by a return to baseline. No spontaneous recurrent seizures are seen, unlike with intraperitoneal, intra-amygdala, and intrahippocampal administration of KA (11, 33). Interestingly, we found male SWELL1 cKO mice exhibited significantly increased seizure severity compared with vehicle-treated littermate controls, as assessed by the modified Racine scale (Figure 1, F and G). There were also nonsignificant trends toward higher rates of severe seizures (Racine score ≥ 5) and mortality in the cKO group (Figure 1, H and I). All mortality occurred within 2 hours of KA injection. These findings were replicated using tamoxifen-treated controls lacking either the Cre recombinase or floxed SWELL1 alleles (i.e., SWELL1^(fl/fl)^ CX3CR1^(WT/WT)^ and SWELL1^(WT/WT)^ CX3CR1^(Cre-ER/WT)^), thereby ruling out off-target effects of tamoxifen (Supplemental Figure 1, D–G). Together, these results demonstrate that seizures alter microglial VRAC expression, and abrogating VRAC expression increases the severity of acute seizures in mice, suggesting a bidirectional relationship between seizure activity and microglial VRAC function.

### Transient microglial loss and morphological remodeling in SWELL1 cKO mice.

**Figure 2:**
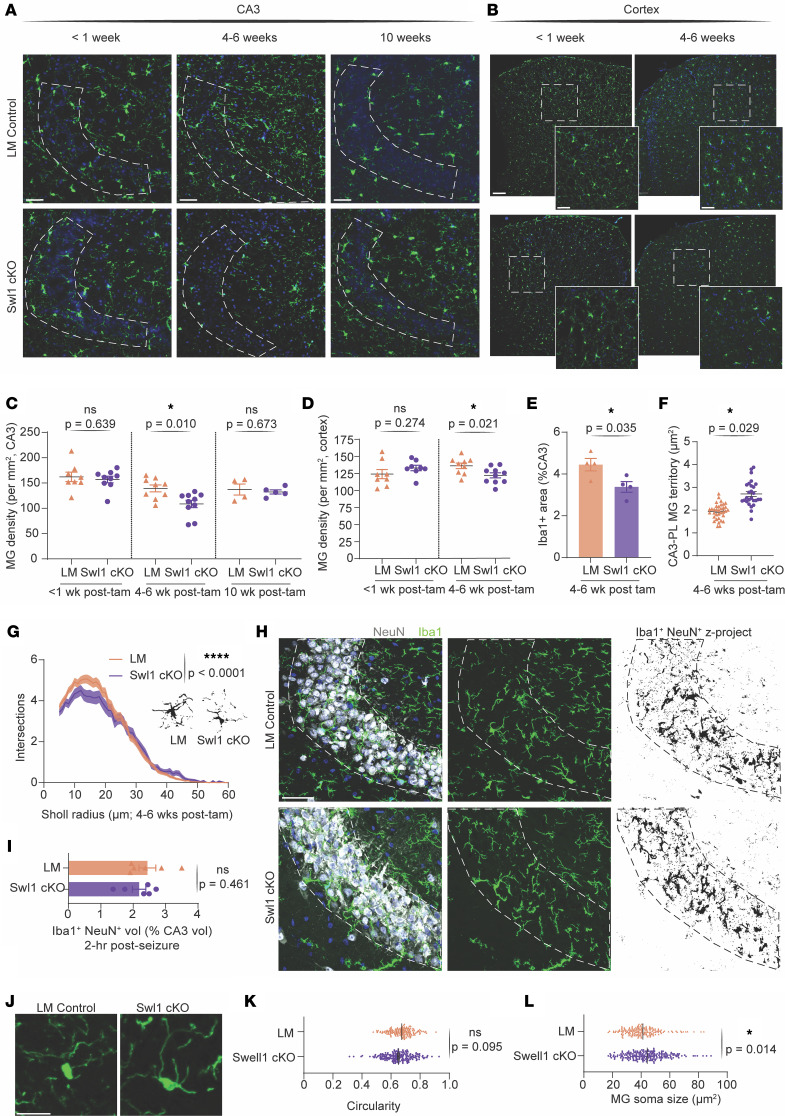
Transient microglial loss and morphological remodeling in SWELL1 cKO mice. (**A**) Microglia density in CA3 pyramidal layer of hippocampus at various time points after the last tamoxifen/vehicle administration (4–10 animals/group/time point examined). Scale bar: 50 μm. (**B**) Microglia density in cortex at various time points after the last tamoxifen/vehicle administration (8–10 animals/group/time point examined). Scale bar: 50 μm. (**C**) Quantification of microglia density in CA3 pyramidal layer (statistic: unpaired *t* test or Mann-Whitney *U* test). (**D**) Quantification of microglia density in cortex (statistic: unpaired 2-tailed *t* test). (**E**) Quantification of Iba1^+^ area in CA3 pyramidal layer (statistic: unpaired 2-tailed *t* test). (**F**) Quantification of microglia territory in CA3 pyramidal layer (dot, individual microglia; 10–40 microglia/mouse, 4–5 mice/group; statistic: nested 2-tailed *t* test). (**G**) Sholl analysis and representative thresholded images of microglia from SWELL1 cKO and LM control mice (10–40 microglia/mouse, 4–5 mice/group; statistic: 2-way ANOVA). (**H**) Microglia (Iba1)–neuron (NeuN) interaction in CA3 pyramidal layer at 2 hours after kainate-induced seizure (6 animals/group examined). Scale bar: 50 μm. (**I**) Iba1-NeuN double-positive volume as a percentage of CA3 pyramidal layer volume (statistic: unpaired 2-tailed *t* test). (**J**) Microglia soma changes at 2 hours after seizure induction (90–160 microglia/mouse, 5–6 mice/group examined). Scale bar: 20 μm. (**K**) Quantification of microglia soma circularity in CA3 (dot, individual microglia; 90–160 microglia/mouse, 5–6 mice/group; statistic: nested 2-tailed *t* test). (**L**) Quantification of microglia soma size in CA3 (dot, individual microglia; 90–160 microglia/mouse, 5–6 mice/group; statistic: nested 2-tailed *t* test). LM, vehicle-treated littermate control; PL, pyramidal layer; Tam, tamoxifen. *P <0.05, ****P <0.001.

Previous studies have implicated VRAC/SWELL1 in cell proliferation and survival (29–31). Consistent with these roles, we observed an approximately 10%–20% reduction in microglial density in both the cortex and the cornu ammonis 3 (CA3) pyramidal layer of SWELL1 cKO mice at 4–6 weeks after tamoxifen induction (Figure 2, A–D, and Supplemental Figure 2, A and B). This reduction was accompanied by increased nearest neighbor distances (NNDs), indicative of altered microglial tiling, and a rightward shift in NND distribution curves (Supplemental Figure 2E). Importantly, microglial density returned to control levels by 10 weeks, confirming the transient nature of this reduction (Figure 2, A and C). These findings were further validated by Cre-ER–driven tdTomato expression (Supplemental Figure 2, C and D) and reduced ionized calcium-binding adapter molecule 1 (Iba1) immunoreactivity at 4–6 weeks (Figure 2E). At the time points associated with reduced microglial density, we also noted changes in microglial morphology. Specifically, microglial territory size was increased (Figure 2F), and Sholl analysis revealed complex alterations in arborization patterns (Figure 2G), suggesting structural remodeling in response to SWELL1 loss.

Because these density and morphological changes coincided with the timing of our seizure behavioral experiments, we hypothesized that reduced microglial-neuronal contact might contribute to heightened seizure severity (Supplemental Figure 2F). To test this, we quantified microglia-neuron interactions 2 hours after seizure induction by calculating the percentage of CA3 voxels co-labeled with microglial markers (Iba1 or P2Y12) and the neuronal marker NeuN using high-resolution confocal microscopy (40× objective; voxel size 0.21 × 0.21 × 0.5 μm³). No significant differences were observed between SWELL1 cKO and control mice (Figure 2, H and I, and Supplemental Figure 2, G and H). However, morphometric analysis revealed larger microglial soma size and a trend toward reduced soma circularity in SWELL1 cKO mice (Figure 2, J–L), suggestive of altered activation states. Notably, soma sizes were reduced from baseline in both genotypes (Supplemental Figure 2, I and J). These morphological changes may be attributed to volume redistribution as microglia extend their processes and form bulbous endings or to a volume loss, raising the possibility that swelling-associated signaling mechanisms could contribute to the seizure phenotype in SWELL1-deficient mice.

### SWELL1 deletion impairs microglial release of neuroactive metabolites.

**Figure 3:**
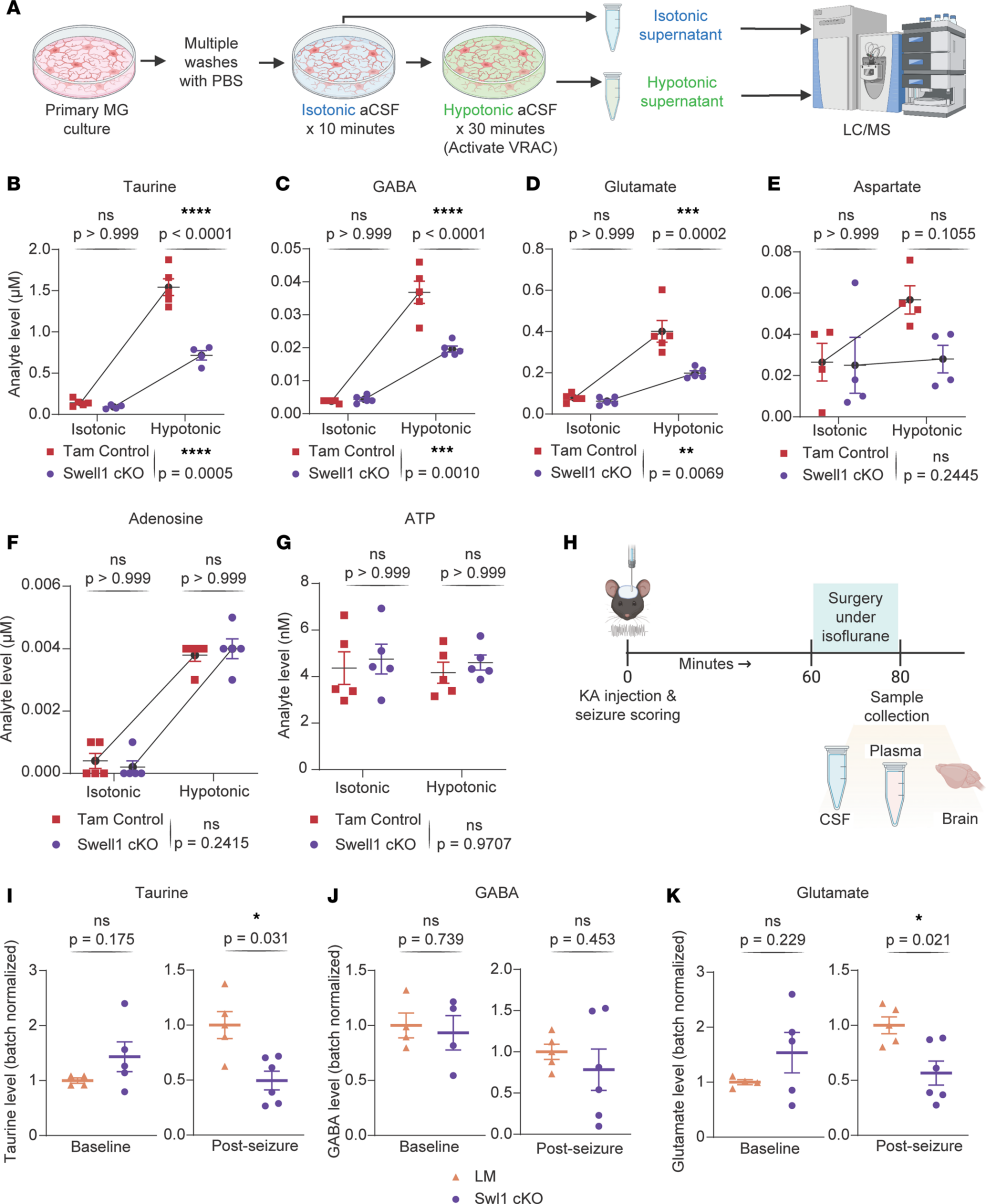
SWELL1 deletion impairs microglial release of neuroactive metabolites. (**A**) Experimental design for activation of SWELL1 (VRAC channel) in purified microglia cultures with a hypotonic stimulus and collection of supernatant for neuromodulator analysis. (**B**–**G**) Quantification of taurine, GABA, glutamate, aspartate, adenosine, and ATP levels in supernatant of SWELL1 cKO versus control microglia maintained in isotonic and hypotonic conditions (dot, 1 well; 4–5 wells/genotype; 50,000 microglia/well; statistic: 2-way ANOVA with Bonferroni’s multiple-comparison test). (**H**) Experimental design to test differences in CSF and plasma metabolites after seizure induction. (**I**–**K**) Batch-normalized taurine, GABA, and glutamate levels (statistic: permutation test). LC/MS, liquid chromatography/mass spectrometry; VRAC, volume regulated anion channel. *P <0.05, **P <0.01, ***P <0.005, ****P <0.001.

VRAC facilitates regulatory volume decrease through the efflux of a variety of organic and inorganic anions, several of which possess neuromodulatory properties (23, 24, 31, 34). Thus, we hypothesized that impaired release of inhibitory metabolites from seizure-activated SWELL1 cKO microglia could contribute to the increased seizure severity observed in this genotype (Supplemental Figure 3C). To test this, we used a mild hypotonic stimulus to activate VRAC in cultured microglia and measured the release of 14 neuroactive metabolites (via liquid chromatography/mass spectrometry [LC/MS]; Figure 3A) and ATP (via colorimetric assay; Supplemental Table 1). Eight metabolites, along with ATP, were reliably detected. Among these metabolites, the release of taurine, GABA, and glutamate was significantly reduced (~50%) in SWELL1 cKO microglia under hypotonic conditions (Figure 3, B–D), whereas the release of aspartate, adenosine, and ATP remained unaffected (Figure 3, D–G).

To determine whether these effects are reflected in vivo, we analyzed cerebrospinal fluid (CSF) samples collected from mice at baseline and 1 hour after KA-induced seizures (Figure 3H). Data were batch-normalized to minimize technical variability. At baseline, CSF levels of taurine, GABA, and glutamate were comparable between genotypes. However, after seizures, SWELL1 cKO mice exhibited significantly lower levels of taurine and glutamate compared with littermate controls (Figure 3, I–K), whereas postseizure GABA levels did not differ significantly (Figure 3J). In both genotypes, postseizure CSF levels of glutamate, GABA, and aspartate were strongly correlated (Supplemental Figure 3, D and E). Notably, taurine levels were highly correlated with these metabolites only in littermate controls, suggesting disrupted taurine regulation in SWELL1 cKO mice. Raw values for all CSF and plasma metabolite measurements are provided in Supplemental Tables 2–5.

### SWELL1 deletion confers paradoxical neuroprotection after seizures.

**Figure 4:**
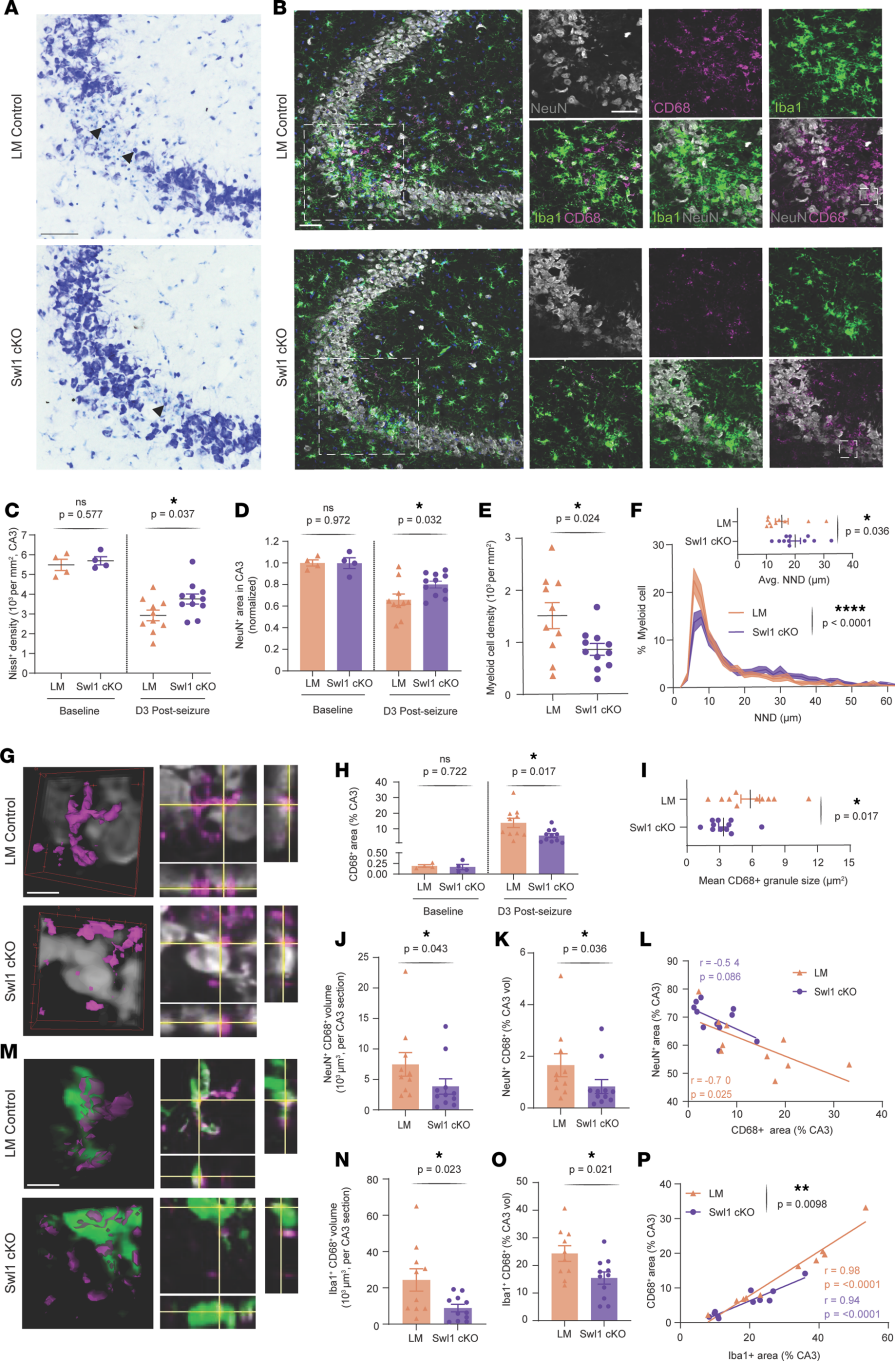
SWELL1 deletion confers paradoxical neuroprotection after seizures due to reduced lysosomal biogenesis. (**A**) Nissl staining showing neuronal loss in CA3 pyramidal layer at day 3 after seizure (arrowheads, pyknotic nuclei; 4–11 animals/group examined). Scale bar: 50 μm. (**B**) Immunofluorescence images showing neuronal loss and myeloid and CD68 responses in CA3 at day 3 after seizure (10–11 animals/group examined). Scale bar: 50 μm. (**C**) Quantification of Nissl-positive cell bodies at baseline and day 3 after seizure (statistic: unpaired *t* test). (**D**) Quantification of relative NeuN-positive area in CA3 at baseline and day 3 after seizure (statistic: unpaired 2-tailed *t* test). (**E**) Quantification of myeloid cell density in CA3 pyramidal layer at day 3 after seizure (statistic: unpaired 2-tailed *t* test). (**F**) Distribution and average of nearest neighbor distances for myeloid cells in CA3 pyramidal layer (statistic: Mann-Whitney *U* test and 2-way ANOVA). (**G**) 3D reconstructed and orthogonal views of NeuN-CD68 volumetric interactions in CA3 pyramidal layer (zoomed-in view of regions of interest [ROIs] shown **B**). Scale bar: 10 μm. (**H**) Quantification of CD68^+^ area in CA3 at baseline and day 3 after seizures (statistic: unpaired *t* test). (**I**) Quantification of mean CD68 granule sizes at day 3 after seizures. (**J** and **K**) Quantification of NeuN-CD68 double-positive voxels in CA3 pyramidal layer (statistic: Mann-Whitney *U* test). (**L**) Correlation between CD68^+^ and NeuN^+^ area in CA3 pyramidal layer at day 3 after seizure (statistic: Pearson’s correlation coefficient). (**M**) 3D reconstructed and orthogonal views of Iba1-CD68 volumetric interactions in CA3 pyramidal layer (zoomed-in view of ROIs shown in **B**). Scale bar: 10 μm. (**N** and **O**) Quantification of Iba1-CD68 double-positive voxels in CA3 pyramidal layer (statistic: unpaired 2-tailed *t* test). (**P**) Correlation between Iba1^+^ and CD68^+^ area in CA3 pyramidal layer at day 3 after seizure (statistic: Pearson’s correlation coefficient, *r*; simple linear regression to compare the slopes). *P <0.05, **P <0.01.

We next assessed the impact of SWELL1 cKO on seizure-induced neuronal loss. Using Nissl staining, we unexpectedly found that SWELL1 cKO mice exhibited significantly reduced neuronal loss in the CA3 region of the hippocampus compared with littermate controls, as assessed 3 days after KA-induced seizures (Figure 4, A and C). This neuroprotection was confirmed with NeuN staining (Figure 4, B and D). Importantly, baseline neuronal density did not differ between genotypes (Supplemental Figure 4, A–D). These findings were paradoxical, as SWELL1 cKO mice experienced more severe seizures.

Given that seizure-induced neurodegeneration is closely linked to microglial phagocytosis, we next examined the microglial and lysosomal responses. We found that Iba1^+^ cells aggregated in the CA3 region in both groups; however, SWELL1 cKO mice showed significantly lower microglial cell density and Iba1 immunoreactivity (Figure 4, B and E, and Supplemental Figure 4, F and G). In line with lower cell density, NNDs were significantly increased in SWELL1 cKO mice, with a rightward shift in the distribution (Figure 4F), indicating more distant microglial tiling. Ki-67 staining was performed to assess whether differential proliferation contributed to less intense microgliosis in SWELL1 cKO animals, revealing no differences between genotypes (Supplemental Figure 4, N and O).

We next examined the lysosome marker CD68 in microglia. We found that SWELL1 cKO mice exhibited a blunted lysosomal response, as evidenced by significantly lower CD68 staining intensity and smaller, less numerous CD68^+^ granules in the CA3 region (Figure 4, B and G–I, and Supplemental Figure 4H). Notably, the NeuN^+^ area in the CA3 pyramidal layer showed a strong inverse correlation with CD68 intensity in littermate controls (Figure 4L), consistent with increased neuronal loss being associated with heightened microglial phagolysosomal activity. A moderate, nonsignificant negative correlation was observed in SWELL1 cKO mice, suggesting a disrupted link between neuronal loss and lysosomal engagement. To further investigate phagolysosomal function, we quantified the volume of double-positive NeuN^+^ CD68^+^ voxels — representing neuronal material in contact with or internalized by lysosomes — using high-resolution confocal imaging (20× magnification; voxel size 0.43 × 0.43 × 1 μm³; Figure 4G). SWELL1 cKO mice displayed a significantly smaller volume of NeuN^+^ CD68^+^ voxels compared with controls (Figure 4, J and K), supporting reduced neuronal clearance as the basis for the observed neuroprotection.

Given that CD68 expression is closely tied to microglial activation and proliferation, we examined whether reduced CD68 staining in SWELL1 cKO mice could be explained by fewer microglia. We quantified the volume of Iba1^+^ CD68^+^ voxels as a proportion of total Iba1^+^ volume. Both the absolute and relative volume of these double-positive voxels were significantly reduced in SWELL1 cKO mice (Figure 4, M–O). Moreover, CD68^+^ and Iba1^+^ areas were positively correlated in both genotypes, but the slope of this relationship was significantly lower in SWELL1 cKO mice (Figure 4P), indicating an impairment in the efficiency of lysosomal biogenesis. The data in Figure 4, L and P, represent averages pooled from 3–5 CA3 sections per animal. Notably, substantial variability in Iba1 and CD68 responses was observed between sections from the same animal, suggesting regional heterogeneity in microglial activation. To account for this, we conducted a secondary analysis treating individual sections as biological replicates. This approach confirmed the strong correlations between CD68^+^ and NeuN^+^ areas (Supplemental Figure 4I) and between Iba1^+^ and CD68^+^ areas (Supplemental Figure 4J), including the reduced slope in SWELL1 cKO mice, thus reinforcing our conclusions regarding the reduced efficiency of lysosomal synthesis in the latter. We also examined differences in inflammatory response between genotypes and found higher levels of IL-1β in SWELL1 cKO animals compared with controls (Supplemental Figure 4 K–M).

### Cell-autonomous differences in number and phagocytic deficits in SWELL1 cKO microglia.

**Figure 5:**
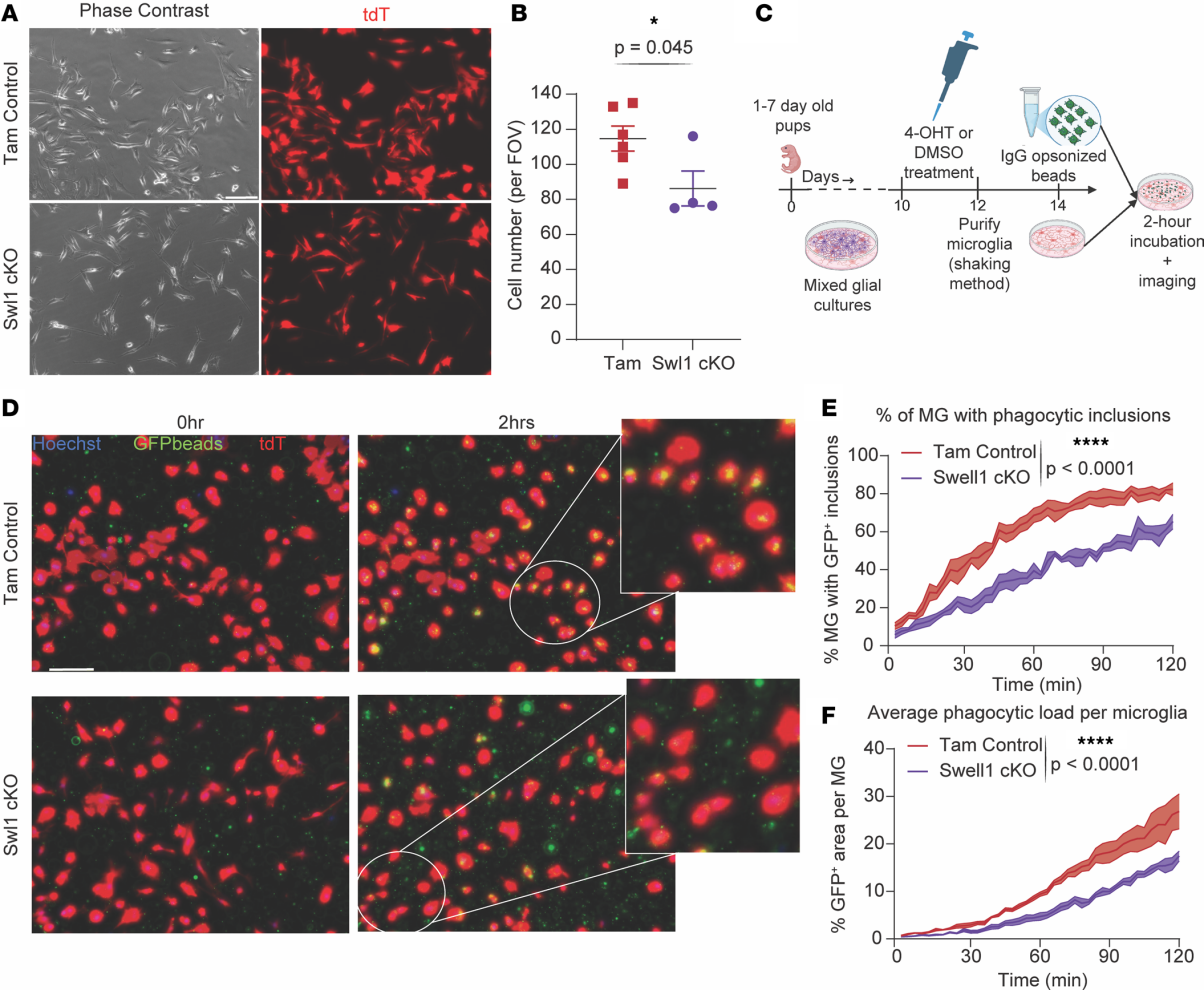
Cell-autonomous proliferative and phagocytic deficits in cultured SWELL1 cKO microglia. (**A**) Microglia density at 10 days in vitro after plating purified microglia at equal density (4–6 wells/genotype examined in a single experiment). Scale bar: 100 μm. (**B**) Quantification of microglia density at 12 days in vitro (statistic: unpaired 2-tailed *t* test). (**C**) Experimental design for in vitro phagocytosis assay with opsonized latex beads. (**D**) Bead phagocytosis assay (4 wells/genotype examined in a single experiment). Scale bar: 100 μm. (**E**) Quantification of percentage of microglia that were positive for phagocytic inclusions over a 2-hour observation window (*n* = 4 wells/genotype; statistic: 2-way ANOVA). (**F**) Quantification of average phagocytic load per microglia expressed as GFP^+^ area (*n* = 4 wells/genotype; results averaged per well; statistic: 2-way ANOVA). ****P <0.001.

To determine whether the differences in cell number and phagocytic impairments observed in SWELL1 cKO mice were cell-autonomous, we performed in vitro experiments using primary cultured microglia. When plated at equal density, SWELL1 cKO microglia exhibited approximately 25% lower cell numbers than tamoxifen-treated controls by day 10 in culture (Figure 5, A and B), indicating a defect in proliferation or survival. Because phagocytosis can be influenced by cell density, we performed the phagocytosis assay at a time point prior to the emergence of significant density differences (Supplemental Figure 5A). GFP^+^ latex beads opsonized with FBS IgG were used as the phagocytic substrate (Figure 5C). Microglia were activated during the assay due to incubation in serum-free medium, which reduces Fcγ receptor saturation (normally caused by serum IgG), thereby enhancing sensitivity to IgG-opsonized beads (Figure 5D). We found that SWELL1 cKO microglia displayed significantly impaired phagocytic kinetics compared with tamoxifen-treated controls, as measured by both the proportion of microglia containing bead inclusions and the average phagocytic load per cell (Figure 5, D–F). These findings were independently confirmed using vehicle-treated, genotype-matched controls (Supplemental Figure 5, B–D). To strengthen the physiological relevance of these findings, we repeated the above assay using apoptotic neuroblastoma cells as phagocytic substrate, reconfirming reduced phagocytosis in SWELL1 cKO microglia (Supplemental Figure 5, E and F). Together, these results demonstrate that the proliferative and phagocytic defects in SWELL1-deficient microglia are intrinsic and cell autonomous.

### Female SWELL1 cKO mice exhibit neuroprotection without increased seizure severity.

**Figure 6:**
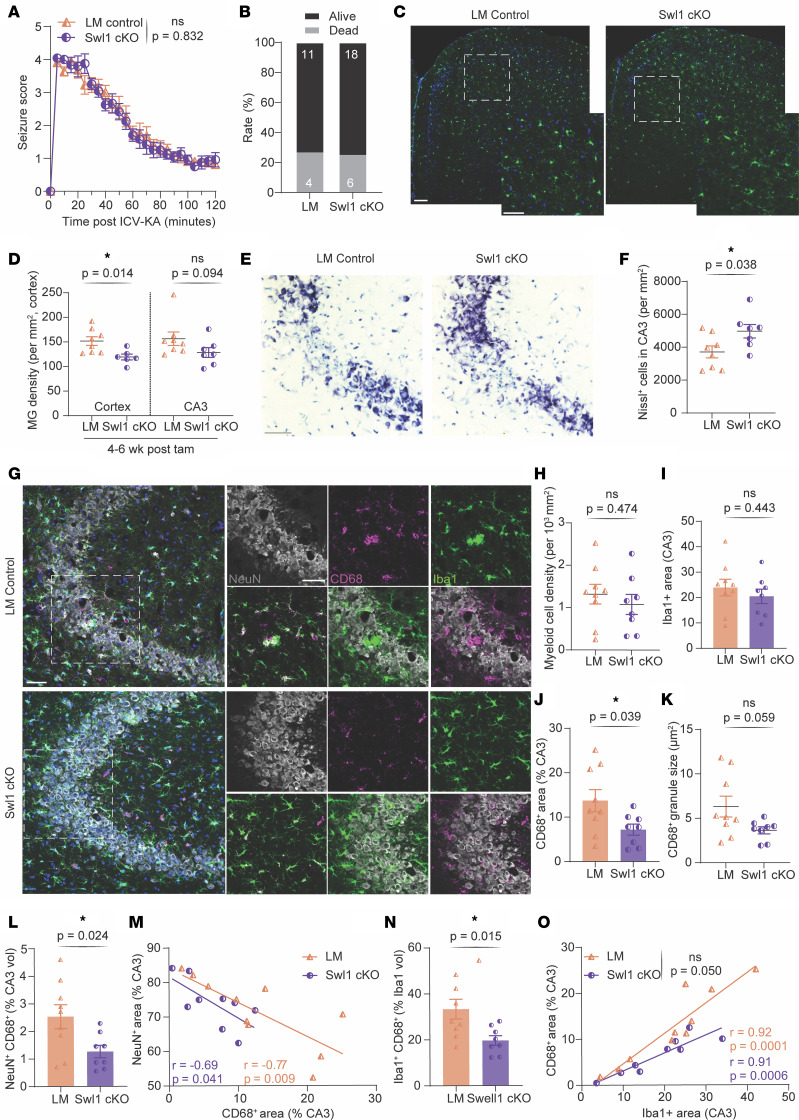
Female SWELL1 cKO mice exhibit neuroprotection without increased seizure severity. (**A**) Longitudinal Racine seizure scores in SWELL1 cKO versus littermate (LM) controls. (*n* = 15–24 mice/group; statistic: 2-way ANOVA). (**B**) Survival rate. (**C**) Microglia density in cortex at 4–6 weeks after the last tamoxifen/vehicle administration (6–8 animals/group examined). Scale bar: 50 μm. (**D**) Quantification of microglia density in cortex and CA3 (statistic: unpaired *t* test and Mann-Whitney *U* test, respectively). (**E**) Nissl staining showing neuronal loss in CA3 pyramidal layer at day 3 after seizure (7–8 animals/group examined). Scale bar: 50 μm. (**F**) Quantification of Nissl-positive cell bodies at day 3 after seizure (statistic: unpaired *t* test). (**G**) Immunofluorescence images showing neuronal loss and myeloid and CD68 responses in CA3 at day 3 after seizure (8–9 animals/group examined). Scale bar: 50 μm. (**H** and **I**) Quantification of myeloid cell density and Iba1^+^ area in CA3 pyramidal layer at day 3 after seizure (statistic: unpaired *t* tests). (**J** and **K**) Quantification of CD68^+^ area and mean granule size in CA3 at day 3 after seizures (statistic: unpaired *t* tests). (**L**) Quantification of NeuN-CD68 double-positive voxels in CA3 pyramidal layer (statistic: unpaired *t* test). (**M**) Correlation between CD68^+^ and NeuN^+^ area in CA3 pyramidal layer at day 3 after seizure (statistic: Pearson’s correlation coefficient). (**N**) Quantification of Iba1-CD68 double-positive voxels in CA3 pyramidal layer as a percentage of Iba1^+^ volume (statistic: unpaired 2-tailed *t* test). (**O**) Correlation between Iba1^+^ and CD68^+^ area in CA3 pyramidal layer at day 3 after seizure (statistic: Pearson’s correlation coefficient; simple linear regression to compare the slopes). *P < 0.05.

Sex differences in microglial function have been reported in the contexts of neuropathic pain, phagocytosis, and P2Y12-dependent behaviors (31, 35–37). Therefore, we examined its role in SWELL1-dependent effects. Interestingly, unlike male SWELL1 cKO mice, females showed no differences in seizure severity or mortality compared with littermate controls (Figure 6, A and B, and Supplemental Figure 6, A and B). Although this trend was noted during early experiments, all female data were analyzed separately to systematically highlight similarities and sex-specific differences.

At 4–6 weeks after tamoxifen administration, SWELL1 cKO females displayed a significant reduction in microglial density in the cortex and a nonsignificant decrease in the CA3 pyramidal layer (Figure 6, C and D, and Supplemental Figure 6C), mirroring the pattern seen in males. Furthermore, like males, female SWELL1 cKO mice exhibited reduced neuronal loss in CA3 3 days after KA-induced seizures (Figure 6, E and F). However, unlike males, female SWELL1 cKO mice did not show significant differences in the overall microglial response, as measured by Iba1^+^ area and cell density (Figure 6, G–I). Despite this, CD68 expression remained reduced in the CA3 region, with significantly lower CD68^+^ area and a nonsignificant reduction in CD68^+^ granule size (Figure 6, G, J, and K). These results indicate that lysosomal activation, rather than general microglial recruitment, may underlie the neuroprotective phenotype in females. Consistent with this interpretation, SWELL1 cKO females showed reduced lysosome-neuron interaction, as evidenced by a lower volume of NeuN^+^CD68^+^ voxels and a moderate (versus strong) negative correlation between NeuN^+^ and CD68^+^ areas (Figure 6, G, L, and M). Additionally, lysosomal synthesis efficiency appeared impaired, as shown by a reduced proportion of Iba1^+^CD68^+^ voxels normalized to total Iba1 volume and a significantly flatter slope in the Iba1-CD68 correlation (Figure 6, G, N, and O). These findings suggest that, as in males, impaired phagolysosomal processing may contribute to neuroprotection in female SWELL1 cKO mice.

To further explore sex-specific effects, we conducted a post hoc analysis comparing neuropathology responses across sexes and genotypes. No significant sex differences were observed in Iba1^+^ or CD68^+^ area in CA3 for either genotype (Supplemental Figure 6, D–F). However, females exhibited less neuronal loss than males, with statistically significant protection in SWELL1 cKO mice and a nonsignificant trend in littermate controls. Additionally, female littermate controls had a higher proportion of CD68^+^ Iba1^+^ voxels (normalized to Iba1^+^ volume) compared with males, despite similar slopes in the CD68-Iba1 correlation (Supplemental Figure 6, G–L). This difference was attributed to a higher intercept, suggesting that at any given level of microglial activation, females produce more lysosomal content. Biologically, this implies that although the rate of CD68 upregulation per unit Iba1 is similar across sexes, female microglia may exhibit a higher baseline lysosomal load.

## Discussion

In this study, we investigated the role of microglial VRAC in acute seizures and postseizure neuropathology, motivated by our observation of seizure-induced alterations in VRAC subunit expression. Using a cKO model targeting the obligate VRAC subunit SWELL1 in myeloid cells, we uncovered 3 major findings: (a) SWELL1 deletion increased seizure severity in male mice, (b) it conferred paradoxical neuroprotection through impaired microglial phagocytosis, and (c) the effects varied by sex, with females displaying preserved neuroprotection despite unchanged seizure susceptibility. These results provide insight into the role of microglial VRAC in modulating neuron-glia interactions in seizures and highlight a mechanistic link between ion channel function, neuroimmune activity, and sex-specific outcomes in epilepsy.

### Methodological considerations in microglial transgenic models for studies of epilepsy.

cKO LRRC8A/SWELL1 models are essential for studying VRAC function, as global KO results in embryonic lethality, early postnatal death, and widespread organ dysfunction (38), underscoring the channel’s fundamental physiological importance. The discovery of the *Lrrc8* gene family and the identification of LRRC8A/SWELL1 as the obligate VRAC subunit (24, 25) have enabled the development of genetic tools to study its cell type–specific and context-specific roles. To date, 4 studies have directly examined microglia-specific VRAC function — 3 using cKO (31, 39, 40) and 1 employing AAV-mediated overexpression (27).

Some of these reports yield conclusions that diverge from both our findings and established VRAC literature in myeloid cells, necessitating careful methodological consideration. Efficient and specific microglial targeting remains challenging, even with recent advances in AAV vector engineering (41). For example, the AAV-mediated overexpression study did not assess transduction efficiency or specificity (27), limiting the interpretability of its results.

The cKO studies have generally used CX3CR1-driven Cre expression to achieve microglia-specific deletion. Although CX3CR1 is the most widely used microglia-targeted Cre driver, it is also active in brain border-associated macrophages (42–44). Additional considerations with this system include the following: (a) the size of the *loxP*-flanked genomic region, which affects the likelihood of spontaneous recombination; (b) potential off-target effects of peripheral *Cre-ER* activation; (c) tamoxifen toxicity; and (d) differences between constitutive and inducible Cre models. When studying microglia in seizure models, 2 other variables are critical: the genetic background of experimental groups and CX3CR1 haploinsufficiency in Cre KI/KO lines. Genetic background can significantly affect seizure susceptibility and severity, even among closely related inbred strains (45, 46). Additionally, *CX3CR1* modulates both seizure severity and seizure-induced neuronal loss (10, 47), and its haploinsufficiency can affect microglial function (10, 48, 49).

In our model, the loxP-flanked region of the Swell1^(fl/fl)^ allele spans approximately 5 kb (50), which minimizes the risk of spontaneous recombination (42, 43). We employed multiple control groups to account for experimental confounders: tamoxifen-injected non-littermate controls for Cre-ER and tamoxifen-specific effects and vehicle-treated littermate controls to account for CX3CR1 haploinsufficiency, genetic background, and residual recombination. Tamoxifen was administered at 5 weeks of age — a developmental time point that does not perturb the microglial transcriptome or morphology (42, 51) — followed by a 3- to 4-week recovery period to allow turnover of short-lived peripheral myeloid cells, thereby limiting recombination to long-lived tissue resident macrophages and microglia (52). We also excluded meninges, choroid plexus, and vascularized regions from our histological analyses to minimize brain border-associated macrophage contamination, although some off-target recombination cannot be entirely ruled out. Future work may benefit from more microglia-specific genetic tools.

### How SWELL1 may influence microglia-neuron communication during acute seizures.

Transcriptomic and proteomic analyses suggest that, at baseline, microglial VRAC may primarily be composed of LRRC8A (SWELL1) and LRRC8D subunits (Figure 1B and Supplemental Figure 1C), consistent with previously published datasets (11, 32, 53, 54). Functional VRAC channels require LRRC8A in combination with at least 1 additional LRRC8 family member, with channel properties and substrate selectivity shaped by subunit composition (23–25, 34). For example, LRRC8D is necessary for the transport of neutral amino acids, such as taurine and GABA, whereas LRRC8B and LRRC8C are dispensable for this function (34).

After seizures, we observed downregulation of *Lrrc8a* and upregulation of *Lrrc8b-d* in microglia (Figure 1, A and B), suggesting a potential shift in VRAC stoichiometry. This may reflect an adaptive response to the evolving demands of the postseizure environment. However, since transcriptomic data were derived from CD11b^+^ populations, these changes could also reflect compositional shifts in myeloid subpopulations. To directly test the functional role of microglial VRAC in seizures, we generated a SWELL1 cKO model in microglia, using an experimental design optimized to minimize peripheral effects and isolate microglia-specific contributions.

SWELL1 cKO males exhibited increased seizure severity (Figure 1, F–I). To investigate underlying mechanisms, we focused on 2 VRAC-associated processes: regulation of cell proliferation and release of neuroactive metabolites (34, 38). We observed a transient reduction in microglial density in the cortex and CA3 region during the time window in which seizures were induced (Figure 2, A–D, and Supplemental Figure 1, D–G). This contrasts with 2 previous studies that found no baseline differences in microglial density in SWELL1 cKO models (31, 39). Potential explanations for this discrepancy include the use of constitutive versus inducible Cre drivers, limited sample size in prior studies, and the transient nature of the effect, which resolved by 10 weeks after tamoxifen treatment in our model.

Microglial depletion has previously been shown to exacerbate seizure severity in the KA model (6, 7), raising the possibility that even a modest (~10%–20%) reduction in microglial density could contribute to heightened excitability. However, we found no differences in microglia-neuron physical interactions 2 hours after seizure (Figure 2, H and I, and Supplemental Figure 2, G and H). This may be due to compensatory morphological changes such as increased microglial territory size (Figure 2F), which could allow fewer microglia to maintain normal neuronal surveillance.

Interestingly, despite an overall reduction in soma size from baseline after acute seizures, SWELL1 cKO microglia were larger than those in controls (Figure 2, J–L). Prior studies have established that VRAC activation triggers the release of various organic and inorganic anions, including inhibitory and excitatory neuromodulators, such as taurine, GABA, and glutamate (24–26, 34, 55). We hypothesized that impaired release of inhibitory metabolites might disrupt the excitatory/inhibitory balance and contribute to increased seizure severity (Supplemental Figure 3C). Release of taurine, GABA, and glutamate was significantly attenuated in SWELL1 cKO microglia (Figure 3, B–D) while ATP was unaffected (Figure 3G), contrasting with reports from BV2 and HeLa cells (31, 56). This discrepancy may reflect differences in cell type, VRAC composition, or activation conditions, such as sphingosine-1-phosphate (S1P) versus hypotonic stress (31, 32, 34, 56). We extended these findings in vivo by analyzing CSF collected from mice before and 1 hour after seizure induction (Figure 3H). Although baseline levels were similar across groups, SWELL1 cKO mice exhibited lower postseizure levels of taurine and glutamate but not GABA (Figure 3, I–K, and Supplemental Tables 2 and 3). Further, dysregulated taurine homeostasis was seen in SWELL1 cKO animals (Supplemental Figure 3, D and E). These changes may reflect loss of microglial SWELL1, differences in seizure severity, or an interaction between the two.

It is important to note that CSF metabolite concentrations may not accurately reflect those in brain interstitial fluid (57), particularly during seizures when microglial processes physically interact with neurons (7, 9, 58). Such proximity could locally concentrate VRAC-released neuromodulators. Taurine, for example, is a weak GABA-A receptor agonist with established anticonvulsant effects (59–62), and its reduction could reduce inhibitory tone. However, glutamate was also reduced in SWELL1 cKO CSF after seizure. A previous study found elevated seizure susceptibility and mortality in mice with brain-wide SWELL1 deletion (under a Nestin-Cre driver) despite reduced tissue glutamate levels (63), suggesting that VRAC disruption may produce complex shifts in excitatory/inhibitory balance. Further, the disparities observed between CSF and in vitro measurement of GABA could reflect true biological differences (e.g., microglial VRAC activation by complex seizure environment vs. hypotonic stimulus) or technical limitations due to dilution and poor spatial resolution of metabolite measurement with CSF. These findings warrant further investigation using real-time, spatially resolved metabolite sensing in intact brain tissue.

### Phagocytic defects in SWELL1-deficient microglia underlie neuroprotection after seizures.

Despite experiencing more severe seizures, SWELL1 cKO mice exhibited reduced neuronal loss in the CA3 region of the hippocampus (Figure 4, A–D). CA3 is particularly vulnerable to KA-induced seizures due to its enrichment in KA-type glutamate receptors and central role in limbic seizure circuitry (11, 12, 16, 64, 65). Previous studies have identified phagoptosis — a form of cell death driven by microglial engulfment of stressed-but-viable neurons — as a key contributor to postseizure neurodegeneration (13, 21, 66). This prompted us to investigate whether altered phagocytic responses in SWELL1 cKO mice could account for the observed neuroprotection.

After seizures, we observed reduced microgliosis in SWELL1 cKO males (Figure 4, B, E, and F), consistent with prior findings in models of neuropathic pain (31). Lack of a difference in the proportion of Ki-67–positive microglia between genotypes suggests that differences in survival rather than proliferation may be responsible for this observation. Lysosomal responses, as assessed by CD68 immunostaining, were also diminished in SWELL1 cKO animals (Figure 4, B and G–I). CD68 is a member of the LAMP protein family and a robust marker of lysosomal expansion after seizure-induced neuronal injury (11–13, 16, 67). NeuN^+^ CD68^+^ voxels — interpreted as neuronal material within or interacting with phagolysosomes — were significantly reduced in SWELL1 cKO mice (Figure 4, J and K), supporting impaired phagocytosis as a driver of neuroprotection. Further, the reduction in lysosomal response in SWELL1 cKO animals was independent of the low myeloid response, as indicated by a lower volume of Iba1^+^CD68^+^ voxels normalized to total Iba1^+^ volume (Figure 4, N and O). Although CD68 and Iba1 areas were strongly correlated in both groups, the slope of this relationship was significantly reduced in SWELL1 cKO mice (Figure 4P and Supplemental Figure 4J), suggesting reduced lysosomal generation per unit of myeloid mass. Together, these data support impaired lysosomal biogenesis as an intrinsic deficit in SWELL1-deficient microglia.

The mechanisms by which VRAC regulates lysosomal biogenesis remain unclear. One possibility is a direct role for VRAC in lysosomal volume regulation. A recent study demonstrated that lysosomal VRAC currents modulate lysosome size and homeostasis in response to cellular stress (68), supporting a potential direct role. Alternatively, the defect may arise from impaired upstream processes, such as phagocytic engulfment, which influence downstream lysosomal load. In vitro studies revealed that the reduction in number and phagocytic defects observed in SWELL1 cKO microglia were cell-autonomous and that engulfment kinetics were reduced in addition to phagolysosomal defects. Collectively, our results indicate that SWELL1 regulates both microglial number and phagocytosis in a cell-intrinsic manner. In the context of seizures, these impairments blunt phagocytosis and thus mitigate secondary neuronal loss — providing a mechanistic explanation for the paradoxical neuroprotection observed in SWELL1 cKO mice despite worsened seizures in male mice.

Whether VRAC-mediated release of taurine, GABA, or glutamate influences microglial phagocytosis remains to be explored in our future investigations. The phagocytic deficits, differences in microgliosis after seizure, and defects in release of metabolites (taurine, GABA, glutamate) are likely pleiotropic effects of the channel, whose interconnection remains to be explored.

### Sex-specific effects of microglial VRAC.

Sexually dimorphic roles of microglia have been described in neuropathic pain and SWELL1 cKO models (31, 35), supporting the plausibility of such interactions in epilepsy. Notably, female SWELL1 cKO mice did not exhibit increased seizure severity (Figure 6, A and B, and Supplemental Figure 6, A and B). Previous studies have demonstrated that microglial depletion exacerbates both acute and chronic seizures in male mice (6, 7), but similar investigations have not been conducted exclusively in females. Although sex differences in KA-induced seizure models are well established (69), the role of microglia in mediating these differences remains largely unexplored. As such, it is unclear whether the sex-dependent effects observed in our model reflect a broader microglial dimorphism or a VRAC-specific phenomenon. One possibility is that taurine release from microglia during seizure may be a male-specific response dependent on VRAC subunit composition, which will be examined in future studies.

With respect to postseizure neuropathology, female SWELL1 cKO mice showed no significant differences in Iba1^+^ microglial responses relative to littermate controls, in contrast to the attenuated microgliosis observed in males (Figure 6, G–I). Prior work suggests that female mice exhibit less robust microgliosis than males after KA-induced seizures (69), and it is possible that our 3-day postseizure time point was too early to detect such changes in females. Nonetheless, our findings raise the possibility that SWELL1 influences microglial proliferation or activation in a sex-dependent manner. Importantly, like males, female SWELL1 cKO mice exhibited reduced CD68 expression, diminished lysosomal biogenesis, and attenuated neuronal loss (Figure 6, E–G and J–O), underscoring a conserved role for VRAC in regulating microglial phagocytic capacity. These findings suggest that SWELL1-dependent modulation of microglial phagocytosis may be a shared mechanism of neuroprotection across sexes, whereas its role in seizure susceptibility may be male-biased. Such differences may inform precision medicine approaches: for instance, microglia-targeted anti-SWELL1 therapies might offer neuroprotective benefits in female patients with epilepsy without increasing seizure risk.

### Future directions.

Our future investigation will focus on identifying the mechanisms responsible for VRAC activation in the ictal and postictal phases. Some of the candidates include osmolarity changes, increased ATP, intracellular calcium, and superoxide production, all of which are known VRAC activators and observed in the ictal phase (70–73). Similarly, during the days and weeks after seizure, S1P may drive VRAC activation. S1P is a known VRAC activator (23, 70) as well as a “find me” phagocytic signal, acting via the S1P receptor family (21). It is possible that some of the phagocytic activity attributable to S1P may be driven by VRAC rather than S1P receptors and needs to be examined. We would also investigate the mechanisms underlying the pleiotropic effects of the channel including metabolite release, phagocytosis, and effect on microglia proliferation/survival.

## Methods

### Sex as a biological variable.

Both sexes were used to account for sex as a biological variable.

### Animals.

SWELL1^(fl/fl)^ mice were provided in-house (see Author contributions). CX3CR1^(Cre-ER/Cre-ER)^ mice (B6.129P2(Cg)-*Cx3cr1^tm2.1(cre/ERT2)Litt^*/WganJ; strain 021160), tdT^(fl/fl)^ mice (B6.Cg-*Gt(ROSA)26Sor^tm14(CAG-tdTomato)Hze^*/J; strain 007914), and WT mice (C57BL/6J; strain 000664) were purchased from The Jackson Laboratory. SWELL1^(fl/fl)^ tdT^(fl/fl)^, SWELL1^(fl/fl)^ CX3CR1^(Cre-ER/Cre-ER)^, and SWELL1^(fl/fl)^ tdT^(fl/–)^ CX3CR1^(Cre-ER/WT)^ (Supplemental Table 6) mice were generated in-house through crossbreeding. They were housed in temperature- and humidity-controlled environments with 12-hour light/12-hour dark cycles and ad libitum access to food and water. To induce gene recombination, tamoxifen (150 mg/kg) or corn oil (vehicle; Supplemental Table 6) was i.p. administered to 5-week-old mice on alternate days for a total of 4 injections. This resulted in 3 groups of experimental animals. The main experimental group was SWELL 1 cKO [SWELL1^(fl/fl)^ tdT^(fl/–)^ CX3CR1^(Cre-ER/WT)^] plus tamoxifen. The littermate control group (aka genetic background control) was SWELL1^(fl/fl)^ tdT^(fl/–)^ CX3CR1^(Cre-ER/WT)^ plus vehicle (corn oil) to control for genetic background and CX3CR1 heterozygosity. The tamoxifen control group of SWELL1^(fl/fl)^ tdT^(fl/fl)^ or tdT^(fl/–)^ CX3CR1^(Cre-ER/WT)^ plus tamoxifen was used to control for adverse/nonspecific effects of tamoxifen.

Seizure experiments were performed at least 3–4 weeks after the last tamoxifen/corn oil injection. The intervals between last tamoxifen/vehicle injection and ICV-KA administration are found in Supplemental Table 7.

### RNA-seq data and analysis.

Microglial bulk RNA-seq data recently published by us (11) and publicly available were reanalyzed to assess changes in microglia homeostatic genes, known seizure-related genes, and *Lrrc8* family in WT mice before and 7 days after seizure. Expression changes are presented as log_2_ fold-change or FPKM values, and significance was assessed with *q* values (FDR-adjusted *P* values).

### Microglia isolation from adult brain and qPCR.

Microglia isolation protocol was adapted from Scheyltjens et al. (74). Briefly, mice were deeply anesthetized and perfused with ice-cold PBS, and brains were rapidly removed. Olfactory bulbs and cerebellum were discarded. The remaining cerebrum was cut into fine pieces, transferred to 6-well plates containing 2 mL RPMI media/well, and maintained on wet ice (Supplemental Table 6). Enzymatic (cocktail of DNase I, collagenase I, and collagenase IV) and mechanical (pipette trituration) dissociation of the tissue were performed, followed by straining (70 μm filters) and removal of myelin and debris using a 30% Percoll gradient. Cells were then pelleted with centrifugation, resuspended, and magnetically labeled with the EasySep Mouse CD11b Positive Selection Kit II (STEMCELL Technologies). This kit targets CD11b^+^ cells for positive selection with antibodies recognizing the CD11b surface marker. Following the manufacturer’s instructions, both CD11b-positive (predominantly microglia) and CD11b-negative (astrocytes, oligodendrocytes, few neurons) fractions were collected. The CD11b-positive fraction was used to evaluate SWELL1 cKO efficiency, and the CD11b-negative fraction was used to assess the specificity.

Cells were then lysed using RNeasy Micro kit, and RNA was extracted following the manufacturer’s instructions. RNA quantity was measured with a microvolume spectrophotometer. It was then reverse-transcribed to cDNA using iScript cDNA Synthesis kit following the manufacturer’s protocol. Real-time quantitative PCR for *Lrrc8A* and *Gapdh* were then performed using SsoAdvanced Universal SYBR Green Supermix. Details of all the reagents are in Supplemental Table 6.

### Induction of KA status epilepticus.

Three days prior to their surgery, 9- to 12-week-old mice were provided with ibuprofen (0.2 mg/mL) analgesia in their drinking water. They were induced with 3% isoflurane and maintained on 1.5%–2% isoflurane with oxygen flow during surgery. Skull was exposed and a 0.9–1 mm wide hole was stereotactically drilled, centered at 1 mm lateral and 0.2 mm posterior to bregma on the left side. A 6 mm long, 25 G stainless steel cannula (Supplemental Table 6) was then implanted in the left lateral ventricle with its bottom at 2.05 mm below the skull surface. The cannula was sealed in place and the incision was covered with layered application of iBond and dental cement (Supplemental Table 6) and cured with UV light for 1 and 2 minutes, respectively. Ibuprofen water was discontinued 3 days after surgery. On day 4, 0.1–0.12 μg KA (Supplemental Table 6) dissolved in 4–5 μL of PBS was injected through the cannula to induce acute seizure. The mice were then placed in clear cylindrical buckets and observed for 2–3 hours. All seizure experiments, except a few early cohorts, were recorded using a GoPro camera.

A seizure score based on the modified Racine scale of 9 points was assigned every 5 minutes. The following scoring matrix was used (1 point) freezing behavior; (2 points) frozen with tail raised; (2.5 points) tail raised and lying on its side, unable to maintain posture; (3 points) reared up with forepaw and/or head clonus; (3.5 points) forepaw and/or head clonus while lying on the side, unable to maintain posture; (4 points) rearing and falling, severe clonic movement of multiple extremities, or stereotypic running movements with periods of stillness; (5 points) level 4 activity exceeding 15 minutes, vigorous jumping; (6 points) tonic phase arrest (extensor tonic posturing of both upper and lower limbs and ventral flexion of tail) with brief cessation of respiration followed by spontaneous return to fast breathing; (7 points) tonic phase arrest with irreversible cessation of respiration and death. Only mice that achieved a seizure score greater than 3 within the first 30 minutes of ICV-KA administration were included for further analysis. All seizure phenotyping was performed in a genotype-blinded manner to prevent observer bias. Longitudinal and average seizure scores, proportion of mice experiencing at least 1 severe seizure (score ≥ 5), and mortality were compared between genotypes.

### Tissue collection for histopathology.

Mice were deeply anesthetized with isoflurane and sequentially perfused transcardially with 30 mL each of ice-cold PBS and 4% paraformaldehyde (PFA). Brains were rapidly removed and postfixed in 4% PFA for 24 hours followed by 30% sucrose in PBS for at least 4 days for cryoprotection. After freezing in molds with OCT, 20 μm thick coronal sections from anterior to posterior hippocampus were cut using a cryostat and placed on adhesive glass slides. The slides were stored at –20°C until further use. See Supplemental Table 6 for reagents. See Supplemental Methods for details on immunofluorescence staining, cresyl violet staining, and image analysis.

### Primary microglia cultures.

Brains were extracted from 2- to 7-day-old pups (Supplemental Figure 3A). Cerebellum, olfactory bulb, and meninges were removed. The brains were then mechanically and enzymatically digested into single-cell suspension using 0.25% trypsin and filtered through a 100 μm sterile strainer. The resulting mixed glial suspension was cultured in DMEM/F12 media supplemented with 10% FBS and 1% penicillin/streptomycin in T-75 flasks for 12–17 days. Once the astrocytic layer reached confluence and abundant microglia were visualized in the mixed cultures (round, pearly cells loosely adherent to the top of astrocytes or floating in the media), they were treated with 5 μM 4-OHT or vehicle (DMSO) for 48 hours. Three groups were thus formed: the SWELL1 cKO group of SWELL1^(fl/fl)^ tdT^(fl/–)^ CX3CR1^(Cre-ER/WT)^ mice treated with 4-OHT; tamoxifen-treated controls of tdT^(fl/–)^ CX3CR1^(Cre-ER/WT)^ mice treated with 4-OHT; and vehicle-treated controls of SWELL1^(fl/fl)^ tdT^(fl/–)^ CX3CR1^(Cre-ER/WT)^ mice treated with DMSO.

To separate microglia, flasks were shaken at 200 rpm for 30 minutes. Microglia density was counted using a hemocytometer and trypan blue stain, and they were plated in d-lysine–coated 12-well or 24-well culture plates (at density ranging from 30,000 to 50,000 cells per well). Experiments were performed 48 hours after replating purified microglia. Microglia were maintained on astrocyte-conditioned DMEM/F12 media. See Supplemental Table 6 for reagents.

### In vitro metabolomics assay.

Primary microglia cultures were washed 3 times with isoosmotic PBS to remove all traces of the media. They were incubated in isotonic aCSF (140 mM NaCl, 5 mM KCl, 1 mM MgCl_2_, 1 mM CaCl_2_, 10 mM HEPES, and 5 mM glucose; osmolarity was adjusted to be between 300 and 320 mOsm/L, pH adjusted to 7.3–7.4; 350 μL/well of 12-well plate) at 37°C (31). The isotonic buffer was carefully removed to a prelabeled 1.5 mL conical vial, and cells were incubated in a 30% hypotonic aCSF (7 parts of isotonic buffer diluted with 3 parts of distilled water; osmolarity 200–215 mOsm/L, pH 7.3–7.4; 350 μL/well of 12-well plate) for 30 minutes at 37°C to activate VRAC currents. Hypotonic buffer was also removed to a 1.5 mL vial. All vials with samples were spun down at 4,000*g* for 5 minutes to pellet any cells. Supernatants were carefully removed to another set of labeled vials inside a laminar flow cabinet and sealed shut. Samples along with 2 vials each of unused isotonic and hypotonic solutions (negative controls) were stored at –80°C or immediately transported on dry ice to the Mayo Clinic Metabolomics Core for analysis. LC/MS was used to analyze 14 neuromodulator panel metabolites (see Supplemental Methods). Average values of respective isotonic and hypotonic negative controls were subtracted from sample values before statistical comparison. Raw values from a single experiment are listed for all analytes without batch normalization. Colorimetric assay for ATP was performed with some modifications from the above protocol (see Supplemental Methods).

### CSF and plasma collection experiments.

CSF and plasma were collected in different groups of animals at baseline (preseizure) and 1 hour after ICV-KA–induced status epilepticus. Briefly, mice were induced with 3% isoflurane and maintained on 1.5% isoflurane with oxygen flow. The head was flexed forward to expose the back of the neck. A vertical incision was made from just behind the ears to the upper thorax. Neck muscles were carefully dissected to expose the atlantooccipital membrane overlying the cisterna magna. Blood was wiped with sequential application of wet (PBS) and dry sterile cotton tips to prevent any blood contamination. Under a surgical microscope, a sharp tipped pipette prepared from a glass capillary (Supplemental Table 6) was guided toward the atlantooccipital membrane using a micromanipulator. The tip was pointed just lateral to the midline, away from visible blood vessels. The capillary was gently taped at the back to facilitate entry into the cisternal space. Its position was readjusted until clear CSF flow was obtained. Around 2–8 μL of CSF was collected per mouse.

For postseizure CSF collection, 1 hour after ICV-KA administration, mice were removed from observation buckets to an induction chamber prefilled with 3% isoflurane. Thereafter, they were maintained on 1.5% isoflurane and CSF was collected as above with the following changes: CSF was collected only for 5–6 minutes, and total time from induction to the end of CSF collection was kept under 40 minutes to minimize the impact of prolonged anesthesia on CSF metabolites. Any samples with grossly visible blood contamination or a volume less than 1.5 μL were discarded. Acceptable CSF samples were then diluted with PBS in a 1:24 or 1:9 ratio to obtain the minimum volume required for LC/MS analysis as per metabolomics core specifications. After CSF collection, mice were deeply anesthetized by isoflurane, and thoracotomy was performed. Then, 200–300 μL blood was obtained directly from the left ventricle using a 25 G needle and syringe and transferred to vials containing EDTA. This was followed by perfusion and brain collection as described earlier. Vials with blood and CSF were centrifuged at 8,000*g* for 10 minutes to pellet cells. Next, 80–100 μL of yellowish to clear plasma supernatant was carefully pipetted to a 0.5 mL conical vial. Similarly, an available volume of CSF supernatant was pipetted into new vials. They were stored at –80°C until transport to the Mayo Clinic Metabolomics Core for analysis (see Supplemental Methods). PBS used for diluting CSF served as a negative control. Average values of negative controls were subtracted from sample values followed by correction for dilution before statistical comparison. Metabolite values were normalized to controls in each batch to reduce batch-to-batch variability.

### In vitro bead phagocytosis assay.

Aqueous GFP latex beads (Supplemental Table 6) were opsonized by incubating in FBS in a 1:5 v/v ratio for 1 hour at 37°C. This coats the bead with IgG, which enhances phagocytic activity of microglia via the Fcγ receptors. The bead-FBS mixture was diluted with serum-free DMEM/F12 to reach a concentration of 1:5:1994 for beads/FBS/media. It is very important to use serum-free DMEM/F12 for this dilution step as serum IgG would saturate the microglia Fc receptors, significantly reducing phagocytosis of opsonized beads. Primary microglia were washed with warm PBS 2–3 times followed by addition of serum-free DMEM/F12 with opsonized beads. Imaging was performed at 5-minute intervals on a Keyence BZX800 microscope (with added incubator module) or after 1–2 hours in a regular incubator at 20× original magnification. Microglia were visualized using endogenously expressed red tdTomato signal (TRITC filter); the beads were visualized using a GFP filter. Phagocytosis was quantified as the percentage of microglia with phagocytic inclusions and the average phagocytic load per microglia (percentage of individual microglia area positive for GFP).

### Modified regulatory volume decrease assay.

Purified primary microglia were washed 2–3 times with PBS and incubated in 700 μL isotonic aCSF per well of a 12-well plate. Single z-planer phase contrast images were obtained every 2 minutes with a Keyence BZX800 microscope (with added incubator module) at 20× magnification. After 10 minutes of imaging in isotonic aCSF, 300 μL of distilled water was gently added along the margins of wells to create 30% hypotonic solution. Imaging was continued every 2 minutes for another 30 minutes. Soma size was manually quantified using ImageJ (NIH) and compared at various time points between the experimental groups.

### Statistics.

Sample sizes were determined using G*Power 3.1 (75) based on pilot experiments conducted on 5–10 animals per group, aiming to minimize animal usage while ensuring sufficient sample to detect robust effect sizes. FDR-adjusted *P* values (aka *q* values) were reported for RNA-seq data. In general, data from 3–5 hippocampal or cortical sections were averaged per animal to obtain histopathology values for comparison. Normality was assessed (where applicable) using the Shapiro-Wilk test, chosen for its effectiveness for both small and large sample sizes. Normally distributed data were analyzed using 2-tailed unpaired *t* tests; a Mann-Whitney *U* (nonparametric) test was used otherwise. A χ^2^ analysis was used to compare proportions. Two-way ANOVA with Bonferroni’s multiple-comparison test was used for data with more than 2 time points from the same animal or culture sample to compare genotype × time / condition effects. Two-way ANOVA was also used to compare the distribution of properties like Sholl intersections × radius and nearest neighbor distances × percentage of microglia between genotypes. Nested *t* tests were used when individual cell properties like microglia territory sizes were compared in order to account for variation that might be present within individual animals, making the comparison of the main genotype effects more robust. Correlations were examined using Pearson’s *r*. Simple linear regression was used to compare the correlation slopes. CSF metabolite data were normalized to the average of respective littermate controls per experimental batch to correct for batch-to-batch variability. A permutation test was used to compare the normalized CSF metabolite data for a more rigorous accounting of small sample sizes, nonstandard distributions, and residual batch effects not corrected by normalization. Raw values for all metabolites for all experiments and groups are provided in the Supplemental tables. All data in figures are presented as mean ± SEM where applicable.

Experiments were designed based on well-established protocols published in the literature and validated by the authors. All seizure experiments were conducted in a genotype-blinded manner to mitigate potential bias. Image processing and data analysis were automated using ImageJ (NIH) macros. CA3 and cortex ROIs were drawn on a DAPI channel while being blinded to other channels to reduce bias. ROI areas were compared between groups to ensure no differences. Multiple controls were used for all key experiments to account for potential confounders. Raw *P* values are reported for all comparisons up to 3–4 decimal points. All analysis is detailed in the Methods to ensure reproducibility and transparency. A list of all the reagents used is in Supplemental Table 6.

### Study approval.

All experimental procedures were approved by the IACUC at Mayo Clinic, Rochester, Minnesota, USA (protocols A00002290-16-R22 and A00002731-17-R23), and the Center for Laboratory Animal Medicine and Care at UTHealth Houston, Texas, USA (protocols AWC-24-0004 and AWC-24-0046).

### Data availability.

Raw data for the experiments have been made available in the Supporting Data Values file associated with this work.

## Author contributions

ASB contributed to conceptualization, study design, data analysis, manuscript writing, and figure drafting and conducted experiments. AD contributed to conceptualization and study design and conducted experiments. LM, MMP, DK, LH, SJ, RH, and TD conducted experiments and data analysis. ED, FG, MA conducted experiments. TTLN conducted experiments, performed data analysis, and drafted figures. FQ was responsible for the experimental design. ADU was responsible for the study design. DBB contributed to the experimental design and generated SWELL1^(fl/fl)^ CX3CR1^(Cre-ER/Cre-ER)^ and SWELL1^(fl/fl)^ tdT^(fl/fl)^ mice. KH provided initial ImageJ analysis scripts and experimental designs. RS provided SWELL1^(fl/fl)^ mice and aided in conceptualization. LJW contributed to conceptualization, study design, manuscript writing, and funding. Authorship order was determined based on an agreement between the two co–first authors.

## Conflict of interest

The authors have declared that no conflict of interest exists.

## Funding support

This work is the result of NIH funding, in whole or in part, and is subject to the NIH Public Access Policy. Through acceptance of this federal funding, the NIH has been given a right to make the work publicly available in PubMed Central.

- NIH R35NS132326 and R01NS088627 (to LJW).

## Supplementary Material

Supplemental data

Supplemental video 1

Supplemental video 2

Supplemental video 3

Supplemental video 4

Supporting data values

## Version 1 06/22/2026

Electronic publication

## Version 2 08/11/2026

Rongzhuo Hua and Tejaswani Datla added to author list
