# Supplementary material for: Microglial SWELL1 deficiency drives male-specific seizure vulnerability but paradoxical neuroprotection through impaired phagocytosis
Source: JCI Insight. 2026 Jun 22;11(12):e197980. doi: 10.1172/jci.insight.197980 (PMC13313489; doi:10.1172/jci.insight.197980)
Supplement: Supplemental data [file jciinsight-11-197980-s007.pdf]

Microglial SWELL1 Deficiency Drives Male-Specific Seizure Vulnerability but Paradoxical Neuroprotection through Impaired Phagocytosis

Abhijeet S. Barath[#1,2,3,4], Aastha Dheer[#1], Laura Montier[3], Mekenzie M Peshoff[3,6], Emily Dale[1], Flavia Goche[1], Thanh Thanh Le Nguyen[5], Mastura Akter[3], FangFang Qi[1], Dimitris Kleidonas[3], Lauren Harris[3], Sarah Jewanee[3], Anthony D. Umpierre[1], Dale B. Bosco[1], Koichiro Haruwaka[3], Rajan Sah[7], Long-Jun Wu*[1,3]

[#]These authors contributed equally

Supplementary figure and video legends; supplementary tables

Videos S1 and S2: Regulatory volume decrease (RVD) response is reduced in Swell1 cKO microglia.

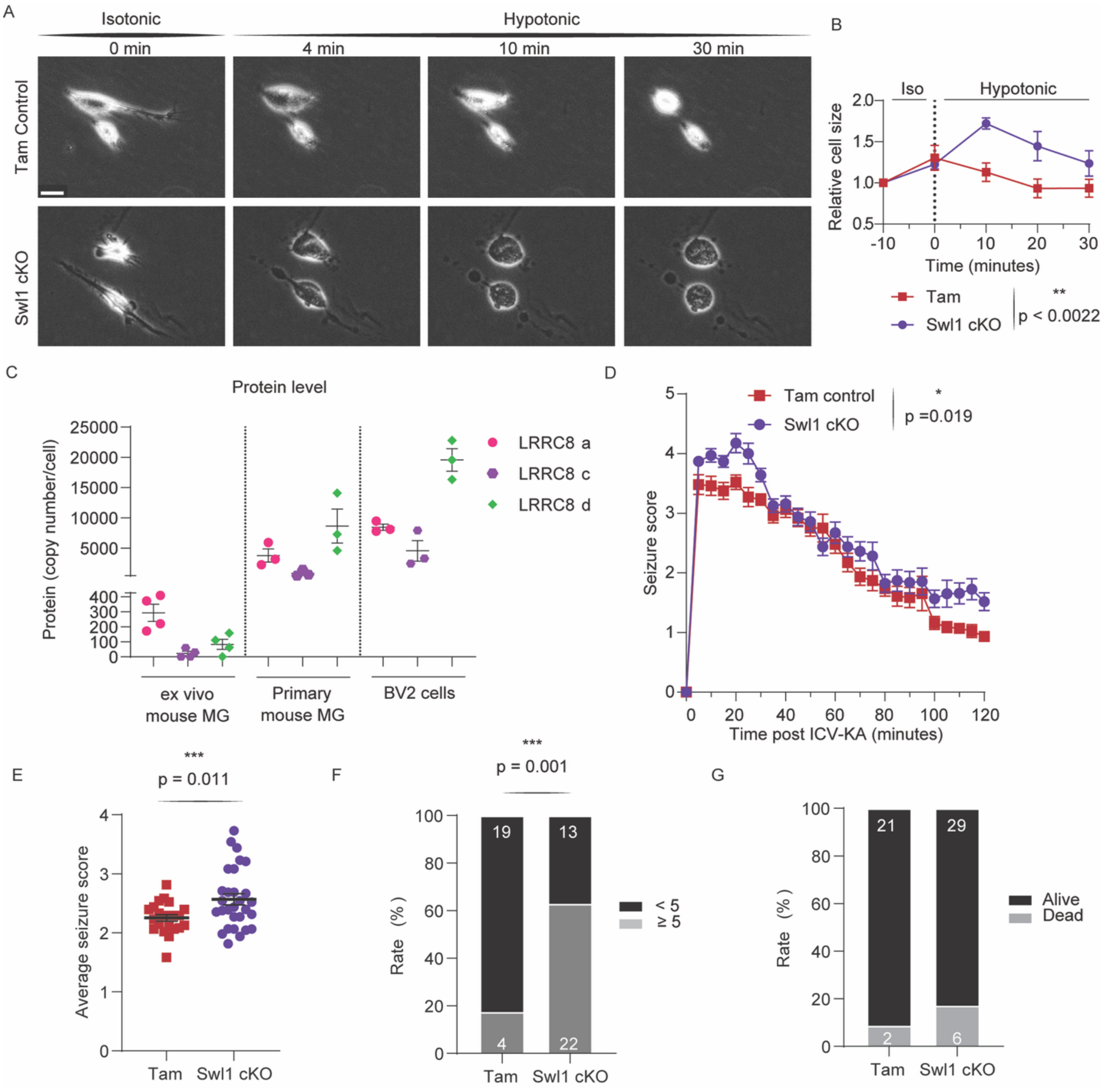

Figure S1. Regulatory volume decrease response is reduced in Swell1 cKO microglia. Swell1 cKO mice display worsened seizure compared to tamoxifen treated controls.

A) Changes in microglia soma size and shape upon exposure to hypotonic medium (5 cells/group examined in a single experiment). Scale bar, 20 µm.

B) Quantification of changes in cell size (n=5 cells per group; statistic: 2-way ANOVA)

C) Baseline protein levels for LRRC8a (Swell1), LRRC8c, and LRRC8d in *ex vivo* mouse microglia, cultured primary microglia, and BV2 cells (data from Lloyd et al., 2024).

D) (D and E) Longitudinal and average seizure scores for Swell1 cKO vs tamoxifen control mice (n = 23-35 mice/ group; statistic: 2-way ANOVA and unpaired t-test)

F) Percent of mice achieving a Racine score of 5 or higher at least once during the two-hour observation period (statistic: Chi-square test)

G) Survival rate.

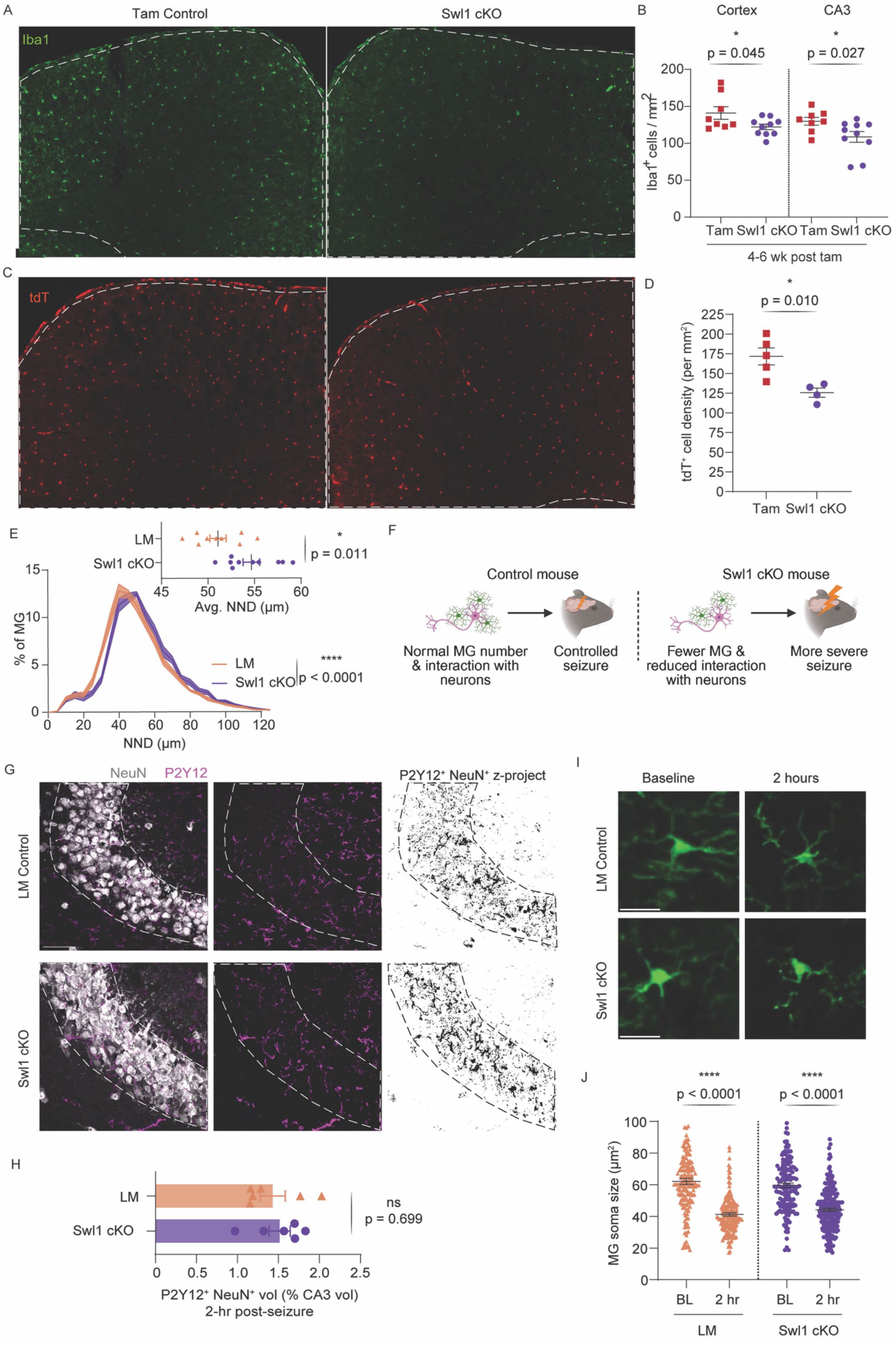
A
Tam Control
Swl1 cKO
Iba1
B
Cortex
CA3
p = 0.045
p = 0.027
Iba1+ cells / mm2
Tam
Swl1 cKO
Tam
Swl1 cKO
4-6 wk post tam
C
tdT
D
p = 0.010
tdT+ cell density (per mm2)
Tam
Swl1 cKO
E
LM
Swl1 cKO
p = 0.011
Avg. NND (µm)
% of MG
LM
Swl1 cKO
p < 0.0001
NND (µm)
F
Control mouse
Swl1 cKO mouse
Normal MG number & interaction with neurons
Controlled seizure
Fewer MG & reduced interaction with neurons
More severe seizure
G
NeuN
P2Y12
P2Y12+ NeuN+ z-project
LM Control
Swl1 cKO
H
LM
Swl1 cKO
ns
p = 0.699
P2Y12+ NeuN+ vol (% CA3 vol)
2-hr post-seizure
I
Baseline
2 hours
LM Control
Swl1 cKO
J
p < 0.0001
p < 0.0001
MG soma size (µm2)
BL
2 hr
BL
2 hr
LM
Swl1 cKO

Figure S2. Microglial Swell1 cKO reduces microglia density but does not affect microglial-neuron physical interaction during acute seizure.

A) Representative images of $Iba1^+$ cells in the cortex (8-10 animals/group examined). Scale bar, 50 μm.

B) Quantification of $Iba1^+$ cells in the cortex and CA3 (statistic: unpaired t-test and Mann-Whitney tests respectively)

C) Representative images of tdTomato positive cells in the cortex.

D) Quantification of tdT^+ cells in the cortex (statistic: unpaired t-test).

E) Distribution and average of nearest neighbor distances for microglia ($Iba1^+$ cells) in cortex.

F) Microglia deficiency hypothesis of Swell1 mechanism in acute seizure

G) Microglia (P2Y12)-neuron (NeuN) interaction in CA3 pyramidal layer at 2 hours after kainate induced seizure (6 animals/group examined). Scale bar, 50 μm.

H) P2Y12-NeuN double positive volume as a percent of CA3 pyramidal layer volume (statistic: Mann-Whitney test)

I) (I and J) Representative images and quantification of changes in soma size at baseline and at 2-hours after seizure. Scale bar, 20 μm. (Statistic: nested t-test; dot, one microglia; 4-6 mice per group, 90-160 microglia per mouse examined)

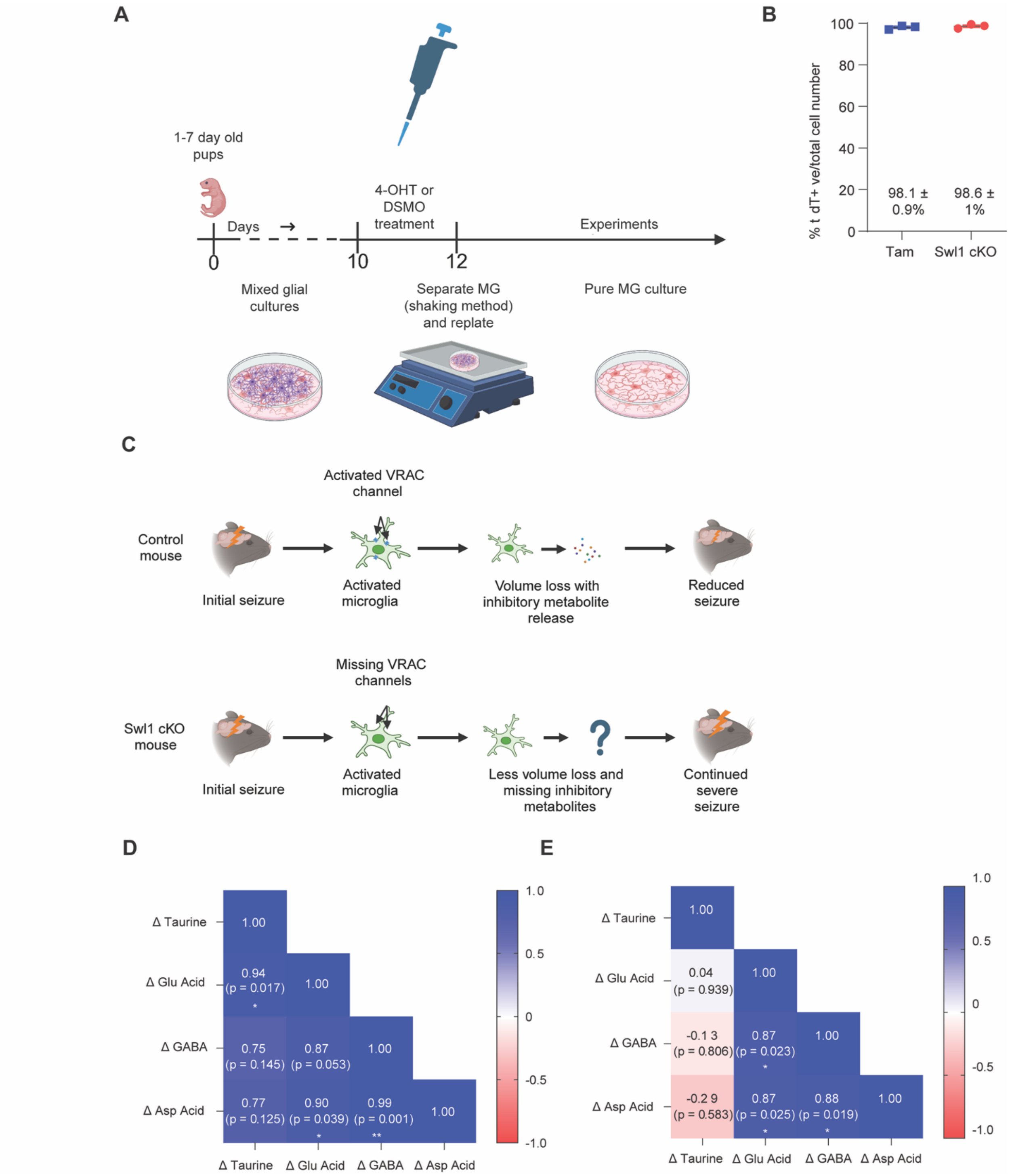

Figure S3. Primary microglia cultures are of high purity. Taurine dyshomeostasis is seen in post-seizure CSF of Swell1 cKO mice.

A) Preparation of purified microglia cultures and 4-OHT treatment *in vitro*

B) Culture purity assessed as % of cells positive for tdTomato signal (3 wells/group examined in a single experiment)

C) Inhibitory neuromodulator hypothesis of Swell1 cKO mechanism in acute seizure

D) (D and E) Correlated changes in CSF metabolites after seizure in littermate controls and Swell1 cKO mice respectively (delta values obtained by subtracting baseline average from post-seizure raw values; statistic: Pearson r).

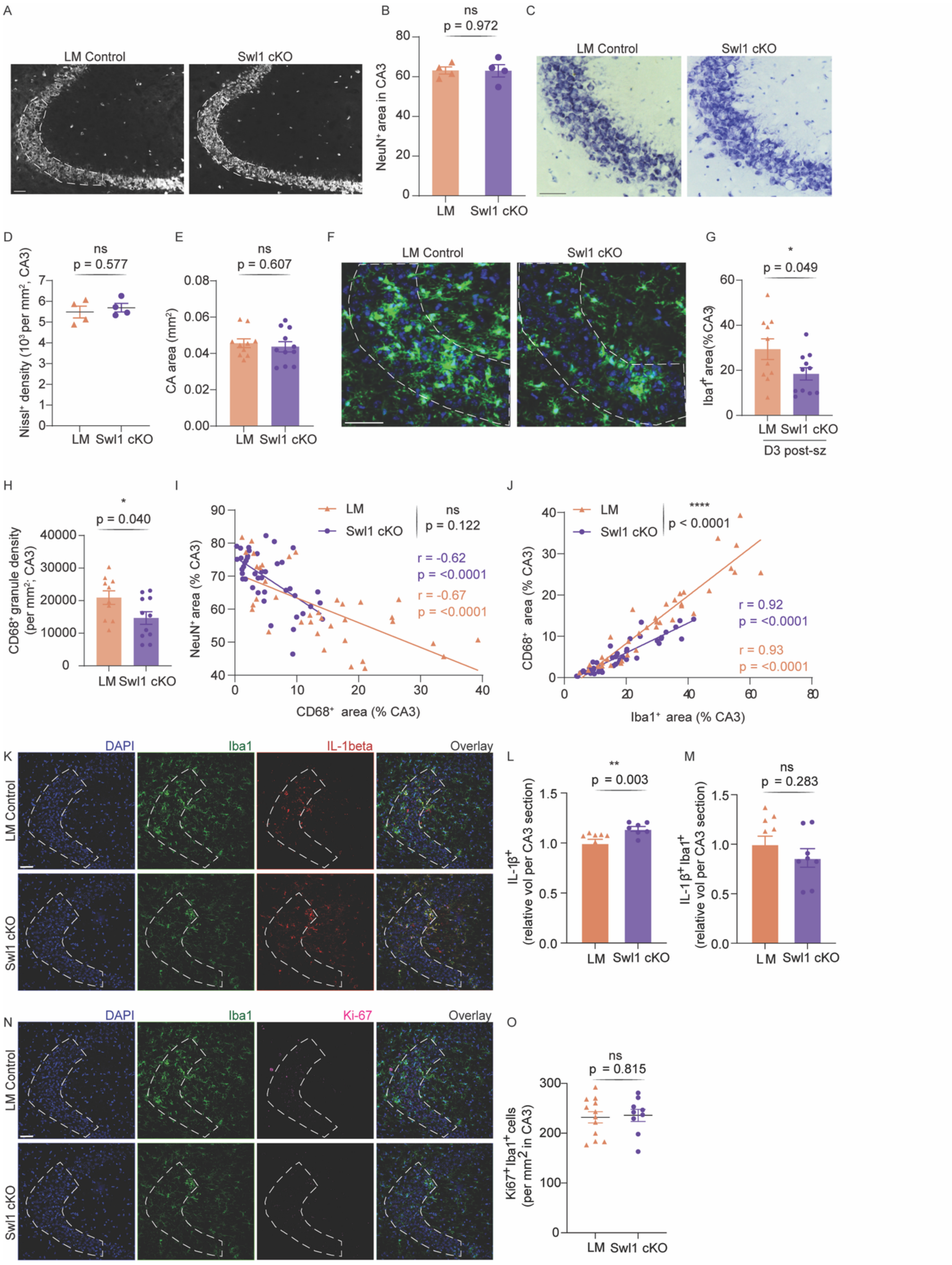

Figure S4. Baseline and post seizure (day 3) neuropathology in male mice.

A) (A and B) Representative images and quantification of NeuN intensity at baseline (4 animals/group; statistic: unpaired t-test). Scale bar, 50 μm.

C) (C and D) Representative images and quantification of Nissl intensity at baseline (4 animals/group; statistic: unpaired t-test). Scale bar, 50 μm.

E) CA3 area measured at 3 days post-seizure (statistic: unpaired t-test).

F) (F and G) Representative images and quantification of Iba1+ area in CA3 at day 3 post-seizure (10-11 animals/group; statistic: unpaired t-test). Scale bar, 50 μm.

H) Quantification of $CD68^+$ granule density in CA3 pyramidal layer (statistic: unpaired t-test).

I) Correlation between $CD68^+$ and $NeuN^+$ area in CA3 pyramidal layer at day 3 after seizure (dot, one CA3 section; 3-5 sections examined per mouse; n = 10-11 mice/ group; statistic: statistic: Pearson correlation coefficient; simple linear regression to compare the slopes)

J) Correlation between $Iba1^+$ and $CD68^+$ area in CA3 pyramidal layer at day 3 after seizure (dot, one CA3 section; 3-5 sections examined per mouse; n = 10-11 mice/ group; statistic: Pearson correlation coefficient; simple linear regression to compare the slopes)

K) Representative images of IL-1β and Iba1 stained hippocampal CA3 at D3 following seizures (7-9 animals/group);. Scale bar, 50 μm.

L) (L and M) Quantification of IL-1β alone and $IL\text{-}1\beta^+$ $Iba1^+$ area in CA3 pyramidal layer (statistic: Mann-Whitney and unpaired t-test respectively).

N) Representative images of Ki67 and Iba1 stained hippocampal CA3 at D3 following seizures (10-12 animals/group);. Scale bar, 50 μm.

O) Quantification of $Ki67^+$ $Iba1^+$ myeloid cells in CA3 pyramidal layer (statistic: unpaired t-test).

Video S3 & S4. Phagocytosis of opsonized beads is reduced in Swell1 cKO microglia.

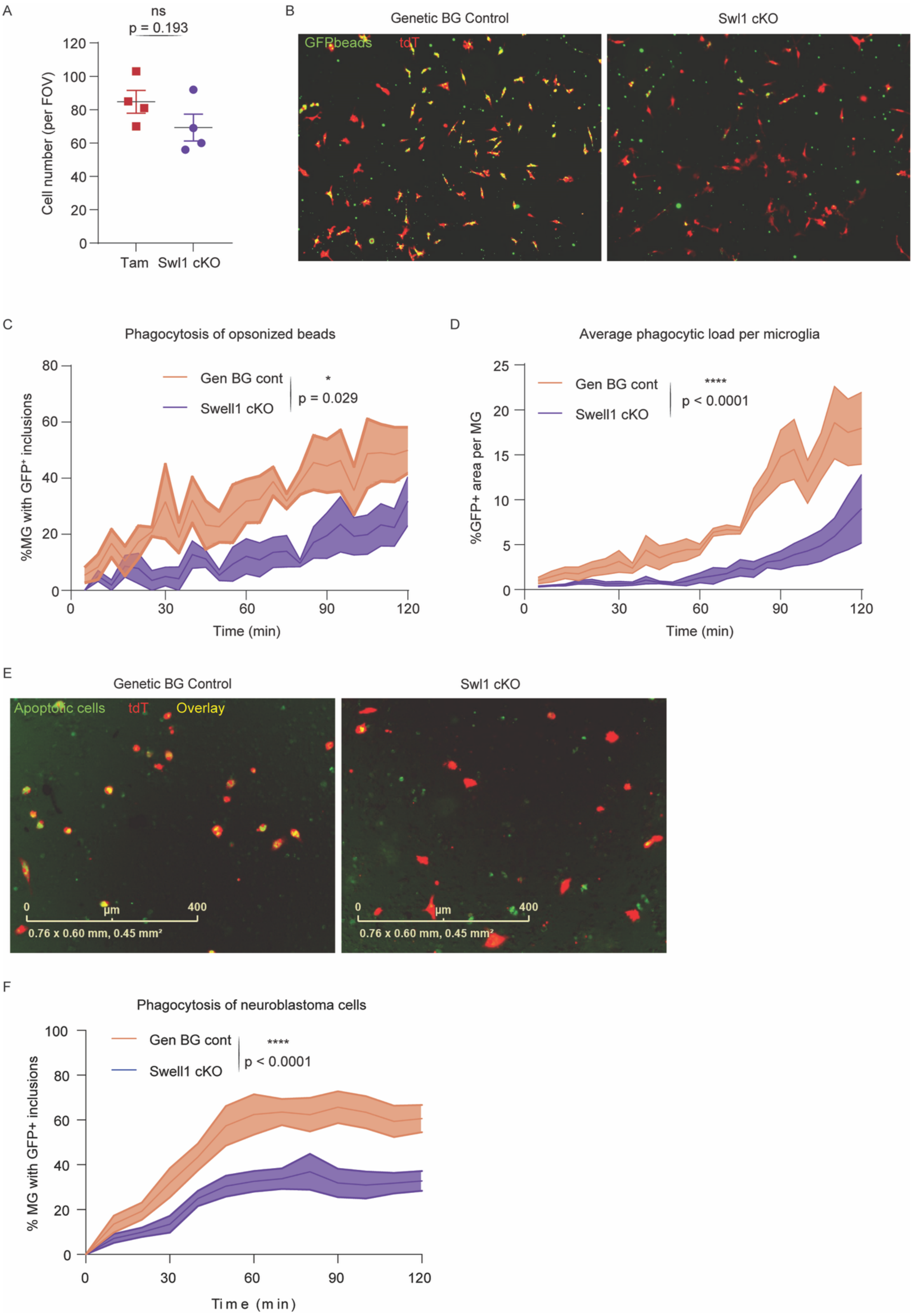
A
ns
p = 0.193
Cell number (per FOV)
Tam
Swl1 cKO
B
Genetic BG Control
Swl1 cKO
GFPbeads
tdT
C
Phagocytosis of opsonized beads
Gen BG cont
Swell1 cKO
*
p = 0.029
%MG with GFP⁺ inclusions
Time (min)
D
Average phagocytic load per microglia
Gen BG cont
Swell1 cKO
****
p < 0.0001
%GFP+ area per MG
Time (min)
E
Genetic BG Control
Swl1 cKO
Apoptotic cells
tdT
Overlay
0.76 x 0.60 mm, 0.45 mm²
F
Phagocytosis of neuroblastoma cells
Gen BG cont
Swell1 cKO
****
p < 0.0001
% MG with GFP+ inclusions
Time (min)

1

Figure S5. Phagocytic defects were confirmed in genotype matched cultured primary microglia.

A) Cell counts at the time of bead phagocytosis assay shown in Fig. 5, D to F.

B) Representative images of bead phagocytosis assay with genetic background matched controls (3-4 wells/genotype examined in a single experiment).

C) Quantification of percent microglia that are positive for phagocytic inclusions over a two-hour observation window (n = 3-4 wells/genotype; statistic: 2-way ANOVA)

D) Quantification of average phagocytic load per microglia expressed as GFP^+ area (n = 3-4 wells/genotype; results averaged per well; statistic: 2-way ANOVA)

E) Representative images of apoptotic cell phagocytosis assay with genetic background matched controls (2 wells/genotype, 5 regions of interest/well examined in a single experiment).

F) Quantification of percent microglia that are positive for phagocytic inclusions over a two-hour observation window (n = 2 wells/genotype, 5 regions of interest/well; statistic: 2-way ANOVA).

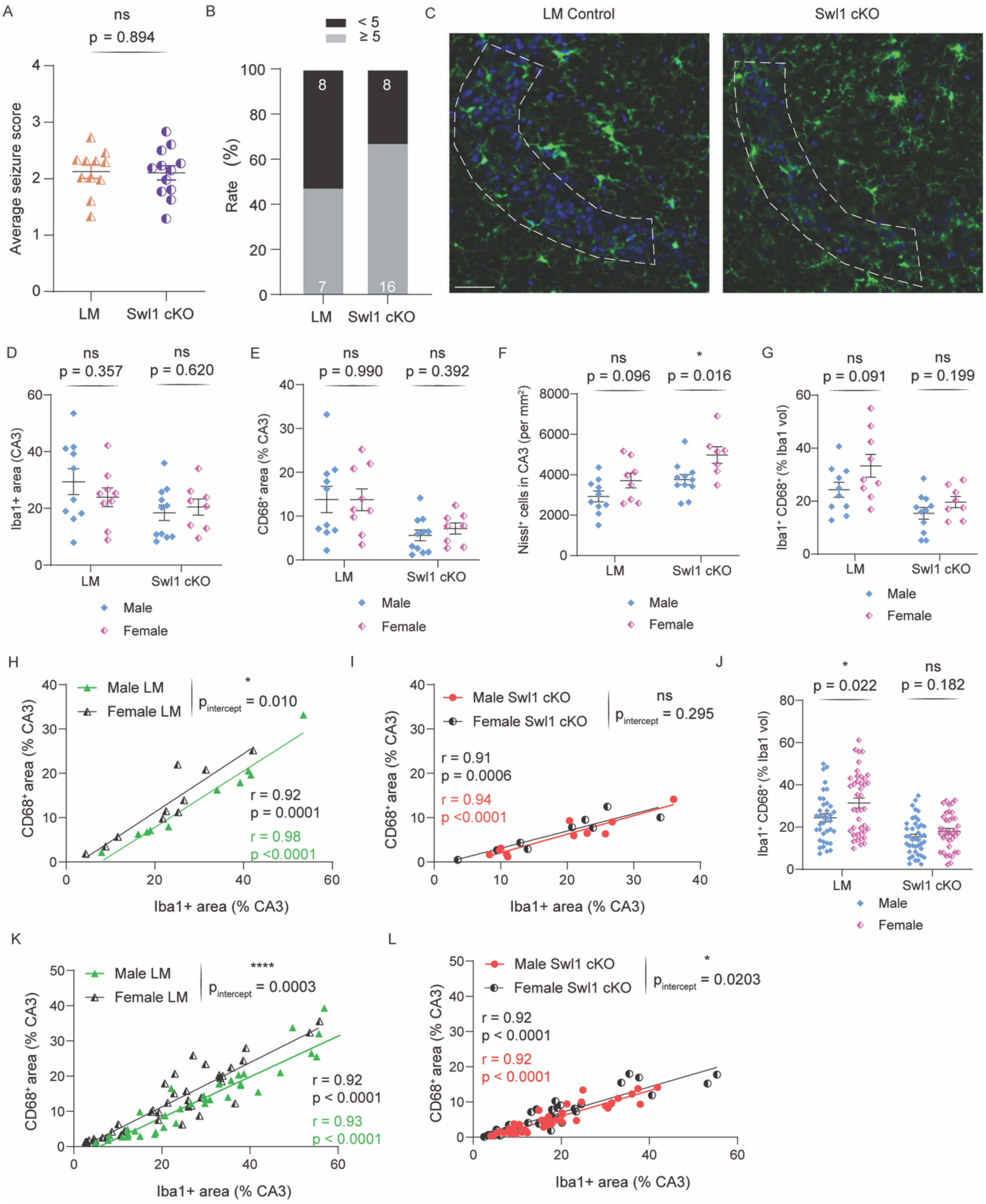

Figure S6. Female Swell cKO mice show no difference in seizure severity and a distinct neuropathology profile.

A) Average Racine seizure scores over the two-hour observation period. (statistic: unpaired t-test)

B) Percent of mice achieving a Racine score of 5 or higher at least once.

C) Representative images of CA3 at baseline showing Iba1+ cells (8-9 animals/group examined). Scale bar, 50 μm.

D) (D to F) Iba1+ area, CD68+ area, and Nissl+ cell density in CA3 at day 3 post-seizure (dot, one animal; statistic: unpaired t-tests)

G) Quantification of Iba1-CD68 double positive voxels in CA3 pyramidal layer as a percent of Iba1+ volume (dot, one animal; statistic: unpaired t-tests)

H) (H and I) Sex wise correlations between $Iba1^{+}$ and $CD68^{+}$ areas in CA3 pyramidal layer at day 3 after seizure in LM and Swell1 cKO animals respectively (dot, one animal; statistic: Pearson correlation coefficient (r) and two-tailed *p*-value; simple linear regression to compare the slopes and intercept)

J) (J to L) Same analysis as from G to I but with individual sections as data points (dot, one CA3 section; 3-5 sections examined per mouse; n = 9-10 mice/ group)

Supplementary tables

Table S1: Metabolites values (in µM; mean ± SD) for primary microglia culture supernatants (n = number of wells)

		**Tam controls**		**Swell1 cKO**	
	**Metabolite**	**Isotonic (n = 5)**	**Hypotonic (n = 5)**	**Isotonic (n = 5)**	**Hypotonic (n = 5)**
1	Acetylcholine	<LLOD	<LLOD	<LLOD	<LLOD
2	Adenosine	<LLOD	0.004 ± 0.001	<LLOD	0.004 ± 0.000
3	Histidine	0.033 ± 0.019	0.025 ± 0.009	0.050 ± 0.034	0.032 ±0.010
4	Serine	0.114 ± 0.096	0.050 ± 0.049	0.214 ± 0.110	0.052 ± 0.045
5	Taurine	0.142 ± 0.043	1.543 ± 0.227	0.091 ± 0.021	0.716 ±0.113
6	Glutamine	0.193 ± 0.075	0.141 ± 0.057	0.214 ± 0.094	0.130 ± 0.058
7	Glycine	<LLOD	<LLOD	<LLOD	<LLOD
8	Aspartic Acid	0.026 ± 0.018	0.057 ± 0.038	0.025 ± 0.027	0.028 ± 0.013
9	Glutamic Acid	0.078 ± 0.020	0.401 ± 0.117	0.063 ± 0.019	0.198 ± 0.026
10	GABA	0.004 ± 0.000	0.037 ± 0.008	0.005 ± 0.001	0.020 ± 0.002
11	Norepinephrine	<LLOD	<LLOD	<LLOD	<LLOD
12	Dopamine	<LLOD	<LLOD	<LLOD	<LLOD
13	Epinephrine	0.018 ±0.005	0.026 ± 0.006	0.025 ± 0.011	0.023 ± 0.003
14	Serotonin	<LLOD	<LLOD	<LLOD	<LLOD

LLOD: Lower limit of detection

Note: If 50% or more values were below LLOD for an analyte in any group, they were discarded from analysis.

Table S2: CSF metabolites values (in µM; mean ± SD) for male mice (n = number of mice)

		**Littermate control**		**Swell1 cKO**	
	**Metabolite**	**Baseline (n = 4)**	**Post-sz (n = 5)**	**Baseline (n = 5)**	**Post-sz (n = 6)**
1	Adenosine	<LLOD	<LLOD	<LLOD	<LLOD
2	Histidine	7.50 ± 0.81	11.2 ± 3.76	8.12 ± 2.39	6.27 ± 3.63
3	Serine	33.87 ± 6.00	40.34 ± 18.47	29.86 ± 1.32	26.18 ± 3.48
4	Taurine	66.50 ± 6.38	138.2 ± 79.04	95.31 ± 40.35	80.79 ± 29.05
5	Glutamine	508.2 ± 68.4	771.9 ± 588.3	615.6 ± 173.2	515.1 ± 174.0
6	Glycine	8.22 ± 1.70	17.22 ± 6.88	8.74 ± 3.21	12.53 ± 6.73
7	Aspartic Acid	1.50 ± 0.55	4.49 ± 3.25	1.82 ± 1.73	3.96 ± 1.82
8	Glutamic Acid	3.95 ± 0.37	11.10 ± 4.61	6.07 ± 3.25	7.23 ± 2.66
9	GABA	0.04 ± 0.01	0.11 ± 0.27	0.03 ± 0.01	0.21 ± 0.19
10	Norepinephrine	<LLOD	<LLOD	<LLOD	<LLOD

LLOD: Lower limit of detection

Note: If 50% or more values were below LLOD for a group, those analytes were discarded from analysis.

Table S3: CSF metabolites values (8 in µM; mean ± SD) for female mice (n = number of mice)

		**Littermate control**		**Swell1 cKO**	
	**Metabolite**	**Baseline (n = 4)**	**Post-sz (n = 3)**	**Baseline (n = 5)**	**Post-sz (n = 2)**
1	Adenosine	0.13 ± 0.12	<LLOD	0.07 ± 0.05	0.28 ± 0.21
2	Histidine	6.16 ± 6.81	5.68 ± 2.81	6.41 ± 3.77	10.69 ± 6.49
3	Serine	32.57 ± 30.94	20.75 ± 3.90	31.91 ± 19.33	48.63 ± 26.17
4	Taurine	72.19 ± 52.35	60.03 ± 42.90	82.40 ± 22.49	115.8 ± 90.8
5	Glutamine	551.9 ± 538.0	421.7 ± 93.6	617.3 ± 430.6	961.6 ± 511.8
6	Glycine	5.28 ± 1.86	8.78 ± 4.70	8.35 ± 4.36	5.90 ± 3.69
7	Aspartic Acid	2.38 ± 0.59	3.45 ± 1.59	2.33 ± 1.11	2.40 ± 1.35
8	Glutamic Acid	4.25 ± 0.75	6.73 ± 3.56	4.28 ± 1.37	4.40 ± 2.62
9	GABA	0.15 ± 0.03	0.17 ± 0.13	0.08 ± 0.04	0.09 ± 0.03
10	Norepinephrine	<LLOD	<LLOD	<LLOD	<LLOD

Table S4: Plasma metabolites values (in µM; mean ± SD) for male mice (n = number of mice)

		**Littermate control**		**Swell1 cKO**	
	**Metabolite**	**Baseline (n = 4)**	**Post-sz (n = 6)**	**Baseline (n = 5)**	**Post-sz (n = 6)**
1	Adenosine	0.24 ± 0.15	0.13 ± 0.11	0.13 ± 0.08	0.11 ± 0.06
2	Histidine	116.8 ± 50.4	127.2 ± 29.3	103.9 ± 18.2	107.6 ± 17.27
3	Serine	205.8 ± 68.3	200.5 ± 36.3	179.8 ± 24.29	169.7 ± 57.0
4	Taurine	836.8 ± 304.5	891.7 ± 376.6	639.2 ± 114.5	552.7 ± 182.5
5	Glutamine	946.4 ± 444.5	870.2 ± 187.7	775.8 ± 66.2	689.2 ± 134.4
6	Glycine	490.2 ± 84.5	453.0 ± 112.9	435.5 ± 79.6	295.5 ± 74.4
7	Aspartic Acid	6.83 ± 4.84	10.19 ± 5.94	3.71 ± 1.59	7.84 ± 3.99
8	Glutamic Acid	77.55 ± 30.16	106.6 ± 92.9	31.94 ± 10.38	35.11 ± 22.54
9	GABA	0.55 ± 0.17	1.14 + 1.10	0.27 ± 0.06	0.52 ± 0.75
10	Norepinephrine	0.10 ± 0.01	0.12 ± 0.07	0.10 ± 0.01	0.10 ± 0.06

Table S5: Plasma metabolites values (in µM; mean ± SD) for female mice (n = number of mice)

		Littermate control		Swell1 cKO	
	**Metabolite**	**Baseline (n = 6)**	**Post-sz (n = 3)**	**Baseline (n = 6)**	**Post-sz (n = 2)**
1	Adenosine	0.11 ± 0.02	0.23 ± 0.05	0.11 ± 0.02	0.13 ± 0.01
2	Histidine	124.1 ± 16.3	117.6 ± 35.5	128.7 ± 24.1	151.7 ± 73.7
3	Serine	225.2 ± 34.4	207.7 ± 39.6	201.3 ± 27.3	300.0 ± 154.8
4	Taurine	781.4 ± 179.9	853.1 ± 125.0	757.3 ± 203.7	915.5 ± 202.5
5	Glutamine	889.8 ± 91.0	866.1 ± 251.8	849.1 ± 154.1	888.9 ± 328.7
6	Glycine	341.3 ± 81.54	428.0 ± 18.3	313.7 ± 65.5	455.2 ± 235.0
7	Aspartic Acid	13.20 ± 4.19	26.16 ± 12.76	10.44 ± 5.55	25.33 ± 9.56
8	Glutamic Acid	38.30 ± 10.38	78.92 ± 8.17	28.70 ± 13.84	52.71 ± 5.69
9	GABA	0.15 ± 0.05	0.50 ± 0.27	0.38 ± 0.36	0.19 ± 0.04
10	Norepinephrine	0.07 ± 0.01	0.17 ± 0.10	0.06 ± 0.01	0.07 ± 0.01

Table S6: Resource table

**Reagent or resource**	**Source**	**Identifier/ Reference**
**Animals / strains**		
Swell1$^{(fl/fl)}$ mice	Dr. Rajan Sah's lab, Washington University, St. Louis	PMID: 28436964
tdT$^{(fl/fl)}$ mice (B6.Cg-*Gt(ROSA)26Sor*$^{tm14(CAG-tdTomato)Hze}$/J)	Jackson lab	Strain # 007914
CX3CR1$^{(CreER/CreER)}$ mice (B6.129P2(Cg)-*Cx3cr1*$^{tm2.1(cre/ERT2)Litt}$/WganJ)	Jackson lab	Strain # 021160
WT mice (C57BL/6J)	Jackson lab	Strain # 000664
Swell1$^{(fl/fl)}$:tdT$^{(f/f)}$	Crossed and bred in house	NA
Swell1$^{(fl/fl)}$:CX3CR1$^{(CreER/CreER)}$	Crossed and bred in house	NA
Swell1$^{(fl/fl)}$:tdT$^{(fl/-)}$:CX3CR1$^{(CreER/wt)}$	Crossed and bred in house	NA
**Cell lines**		
TH-MYCN murine neuroblastoma cells	Dr. Eveline Barbieri, Baylor College of Medicine, Houston TX	NA
**Conditional knockout reagents**		

Tamoxifen	ThermoFisher	CAS # 10540-29-1
Corn oil	Fisher scientific	Cat # S25271
**Adult microglia collection for PCR**		
RPMI media	Corning	Cat # 10-040-CV
DNAse I	Worthington	Cat # LS002060
Collagenase I	Worthington	Cat # LS004214
Collagenase IV	Worthington	Cat # LS004209
Adult Brain Dissociation Kit	Miltenyi Biotec	Cat # 130-107-677
EasySep™ Mouse CD11b Positive Selection Kit II	Stemcell technologies	Cat #18970
RNeasy® Micro Kit	Qiagen	Cat # 74004
Microvolume Spectrophotometer (Nanodrop)	Fisher Scientific	Cat # 13-400-525
iScript™ cDNA Synthesis Kit	Bio Rad	Cat # 1708890
SsoAdvanced Universal SYBR Green Supermix	Bio Rad	Cat # 1725271
LRRC8a primer	Integrated DNA technologies (IDT)	Fwd and rev primer sequences: AGCCACAACAACCTGACCTT & TTGTTGCCCAGGTGTAGAGC
GAPDH primer	Integrated DNA technologies (IDT)	Fwd and rev primer sequences: TGCACCACCAACTGCTTAG & GATGCAGGGATGATGTTC

**Seizure model**		
Stainless steel tubing (0.02" OD, 0.004" wall thickness, 0.012" ID)	McMaster Carr	Cat # 89935K91
Terrell Isoflurane, USP	Mayo Inventory Center	Cat # NDC 66794-019
iBond	Patterson Dental	Cat # 74491437
Dental cement (Tetric EvoFlow® Flowable Composite Restorative, 2 g Syringe Refill)	Patterson Dental	Cat # 77172323
Kainic acid	Tocris	Cat # 0222
**Tissue collection for histopathology**		
PBS (10X Phosphate-Buffered Saline, pH 7.4 ± 0.1)	Corning	Cat # 46-013-CM
Formaldehyde (Buffered, Certified, 10% (v/v), 38 to 42g/L, LabChem™)	Fisher Scientific	Cat # LC146705
Sucrose	Millipore Sigma	Cat # S7903-5KG
OCT (Andwin Scientific Tissue-Tek™ CRYO-OCT Compound)	Fisher Scientific	Cat # 14-373-65
Adhesive glass slides (Tissue Path Superfrost™ Plus Gold Slides)	Fisher Scientific	Cat # 22-035813
Cryostat	Leica	Cat # CM1520

**Immunostaining**		
Triton X-100™	Millipore Sigma	Cat # X100-100ML
TBS (10X Tris Buffered Saline)	Fisher Scientific	Cat # 46-012-CM
Normal Donkey Serum	Jackson Immuno Research	Cat # 017-000-121
Goat serum	Millipore Sigma	Cat # G9023-10ML
DAPI Fluoromount-G	Southern Biotech	Cat # 0100-20
Cover glass	VWR International	Cat # 48382-136
**Primary antibodies**		
CD68 (Rat) 1:500	Abcam	Cat # Ab53444
cFos (Rb) 1:500	CST	Cat # 22505
Iba1 (Rb) 1:500	Abcam	Cat # 178847; RRID # AB_2832244
Iba1 (GP) 1:500	Synaptic Systems (SySy)	Cat # 234 308; RRID # AB_2924932
IL-1β (Goat) 1:500	Novus Biologicals	Cat # AF-401-NA
Ki67 (Rabbit) 1:500	Abcam	Cat # Ab15580
NeuN (Rb) 1:500	Abcam	Cat # 177487; RRID # AB_2532109
P2Y12 (Rat) 1:200	Biolegend	Cat # 848002; RRID # AB_2650634
**Primary microglia culture**		

Corning™ Cell Culture Phosphate Buffered Saline (1X)	Fisher scientific	Cat # MT21040CV
Gibco Trypsin-EDTA (0.25%), phenol red	ThermoFisher	Cat # 25200056
Biologix® Cell strainer – mesh size 100 µm	CisNovo	Cat # 15-1100
Gibco DMEM:F12	Thermo Fisher/ Mayo inventory center	Cat # 11320033
Fetal Bovine Serum	Corning	Cat # 35-010-CV
Penicillin-Streptomycin (10,000 U/mL) 100 mL Gibco™	ThermoFisher	Cat # 15140122
Falcon® 75cm² Rectangular Canted Neck Cell Culture Flask with Vented Cap	Corning	Cat # 353136
4-Hydroxytamoxifen Ready Made Solution	Sigma-Aldrich	Cat # SML1666-1ML
Bright-Line hemocytometer	Millipore Sigma	Cat # Z359629
Trypan blue	Corning	Cat # 25-900-CI
300+ kda Poly-D-Lysine Hydrobromide	Millipore sigma	Cat # P7405-5MG
**ATP colorimetric assay**		
Adenosine 5′-triphosphate (ATP) Bioluminescent Assay Kit	Millipore Sigma	Cat # FLAA-1KT

ARL 67156 trisodium salt (ectonucleotidase inhibitor)	Tocris	Cat # 1283
**CSF collection procedure**		
Capillary Glass	Sutter Instruments	Cat # BF100-50-10
**Nissl (Cresyl violet) staining**		
Xylene	Fisher Scientific	Cat # X3P-1GAL
Ethanol, 200 proof (100%)	Fisher Scientific	Cat # 04-355-451
Glacial acetic acid	Milipore sigma	Cat # A6283-100ML
Cresyl Violet acetate	ThermoFisher	Cat # J64318.09
Depex Mounting Medium	Electron Microscopy Sciences	Cat # 13514
***In vitro* phagocytosis assay**		
Latex beads, amine-modified polystyrene, fluorescent yellow-green	Thermo Fischer	Cat # L1030
Fetal bovine serum	Gibco	Cat # A5256701
1% L-Glutamine	StemCell Technologies	Cat # 07100
1% Penicillin-streptomycin	Gibco	Cat # 15070063
pHrodo Green AM Intracellular pH Indicator Dye	Invitrogen	Cat # P35373
pHrodo Labeling Buffer	Sartorius	Cat # 4658

pHrodo Wash Buffer for Incucyte	Sartorius	Cat # 4659
RPMI 1640	Gibco	Cat # 11875093
Staurosporine	Selleck Chemicals	Cat # S1421
Incucyte SX5 Live-Cell Analysis System	Sartorius	
Incucyte 2024B GUI software	Sartorius	

Table S7: Time interval between last tamoxifen/vehicle injection and ICV-KA administration for individual cohort.

**Male mice**		**Female mice**	
**Cohort ID**	**Time interval between last tam/veh injection and ICV-KA (days)**	**Cohort ID**	**Time interval between last tam/veh injection and ICV-KA (days)**
2	24	1	20
4	26	3	25
47	30	6	22
49	25	8	27
50	25	46	28
51	29	48	24
57	31	52	29
58	30	59	37
61	34	65	24
64	24	70	34
67	39		
69	32		

75	38		
78	32		

Appendices

Appendix 1: Tissue staining (Immunofluorescent and cresyl violet) and image analysis

Immunofluorescence staining

Slides were warmed to room temperature and washed with tris buffered saline (TBS). Permeabilization and blocking was performed with 10% donkey or goat serum prepared in 0.4% Triton X-100 in TBS for one hour (Table S6). Slides were then incubated overnight (at least 12 hours) at 4 °C with primary antibodies prepared in 5% donkey or goat serum in 0.4% Triton X-100 solution in TBS. Please see Table S6 for a list of primary antibodies and concentrations used. After overnight incubation, the slides were allowed to warm up to room temperature for an hour followed by 3 washes with 0.1% Triton X-100 prepared in TBS. They were then incubated at room temperature for 1.5-2 hours with corresponding fluorophore conjugated secondary antibodies prepared in 0.4% Triton X-100 solution in TBS. After a few rounds of washing with 0.1% Triton X-100 prepared in TBS slides were counterstained with mounting DAPI and coverslipped. Slides were allowed to dry 24 hours before imaging or storage (-20 °C) for future imaging.

Nissl (Cresyl violet) staining and analysis

Nissl (Cresyl violet or CV) staining was performed to assess density of healthy neurons at baseline at 3 days after seizure. CV stains Nissl substance in the cytoplasm of neurons in PFA or formalin-fixed tissue. Previously cryosectioned 20 µm thick coronal sections were allowed to warm up to the room temperature. Sections we defatted with xylene, hydrated with serially decreasing concentrations of ethanol solutions, washed with tap water, and then incubate with freshly prepared, acidified (pH 3.5-3.8) 0.1% CV solutions for 10 minutes at 37 °C. They were differentiated with a glacial acetic acid solution in 95% ethanol, and brief successions of increasing concentrations of ethanol. Finaly, sections were dehydrated with 100% ethanol,

cleared with xylene and coverslipped using depex mounting media. After drying for 24 hours in a fume hood, imaging was performed with Zeiss Axio inverted microscope, centered on CA3. For analysis using ImageJ, CA3 boundaries were drawn on the image and the number of healthy appearing neuronal somas were marked using the 'multi point' tool. Non-viable neurons identified as small-sized, dense, irregular-shaped pyknotic cells were excluded. Density of viable neurons within the CA3 ROI was computed. Details of all the reagents may be found in Table S6.

Microglia density analysis

To determine the density of microglia, 3-5 sections per region were imaged with Nikon TE2000e fluorescence microscope for each animal. Single z-plane, multi-channel images for were taken with a 10x objective for cortex (FOV: 1331.2 x 1328.6 μm^2) and 20x objective for the CA3 region (FOV: 665.6 x 664.3 μm^2). ImageJ was used to analyze the images using a code with the following logic sequence- (1) Preprocess DAPI: rolling background subtraction with radius 10-20 pixels (larger than the largest foreground object of interest) to suppress autofluorescence; auto threshold with Otsu method. (2) Ask user to draw the boundaries of the region of interest (cortex or CA3 ROI) on a copy of DAPI channel. (3) Preprocess Iba1 channel: 2-step background subtraction- first, suppress any areas with holes (missing tissue) by creating a mask of pixels below the autofluorescence threshold (empirically determined as <3 mean gray value for 8-bit images) and subtract the masked area from main image; second, rolling background subtraction with radius 20-30 pixels to suppress autofluorescence; auto threshold with Otsu method. (4) Image calculator was used to find $DAPI^+$ $Iba1^+$ microglia nuclei on thresholded images using 'AND' function. (5) Area and centroid functions were enabled in the set measurements menu. (6) On the result of image calculator apply the cortex or CA3 ROI and use 'analyze particles' function to obtain a count of $Iba1^+$ $DAPI^+$ particles above 15 um^2 in size. (8) Microsoft excel was used to compute density based on the particle count in the ROI and the ROI area. (9) NND

plugin was used to obtain the nearest neighbor distances based on the centroid values for microglia nuclei. tdTomato channel was analyzed in the same manner as Iba1.

Iba1^{+}, CD68^{+}, and NeuN^{+} area analysis

Sections were imaged and analyzed in the same way as for microglia density analysis with the following changes in the logic sequence- (1) Triangle (instead of Otsu) method was used to threshold the Iba1 channel as it was found to be more sensitive to finer processes. (2) All Iba1 positive areas above the threshold value within the ROI were summed and included for analysis without a size filter. (3) Microsoft excel used to compute % of ROI area positive for Iba1.

Amira Ki67+ Iba1+ analysis

Images were denoised using Nikon NIS Elements software and subsequently analyzed using Amira 3D software (version 2025.1.1, Thermo Fisher Scientific). Images of Iba1 staining were auto-thresholded to obtain a binary label of the cell bodies. Separately, the Iba-1 stained images were passed through a structure enhancement filter, followed by interactive top-hat segmentation, and slightly processed with a line-closing module [to close some gaps] to obtain a binary label of the processes. The two labels were combined to represent the microglia. Separate microglial cells were identified as connected 3D regions with volume > 250 um^3. Images of Ki67 staining were interactively thresholded to define the areas positive for the staining. Microglial cells with at least one voxel that overlaps with Ki67 positivity were identified as Ki67-positive cells. A mask of the CA region of the hippocampus was manually drawn and applied throughout the z-stack. Only the microglial cells with their centers located inside the mask were included in the analysis.

Microglia territory and Sholl analysis

Iba1 stained sections were images with a Zeiss LSM 780 confocal microscope at 20x magnification (FOV: 424.26 x 424.26 μm^2), visually centered on the CA3 region. A z stack volume was then acquired at 1024x1024 pixel resolution with a 1 µm step size across a uniform 10 µm stack. ImageJ was used to analyze the images using a code with the following logic sequence- (1) Iba1 volume was z-projected with 'max-intensity' option and two copies were made. (2) First copy was auto-thresholded with triangle method. (3) Contrast was enhanced in the second copy with saturation of 1% of the pixels for improved visualization of finer processes. (4) DAPI volume was z-projected with 'max-intensity' option and CA3 ROI was drawn and transferred to the contrast enhanced copy of the Iba1 channel. (5) Microglia with somas within the CA3 were identified and boundaries were drawn for their territory (MG territory ROIs). (7) MG territory ROIs were transferred to the thresholded images, and 'multi-measure' operation of the ROI manager was used to determine the territory sizes. (8) An ImageJ plugin(1) was used to calculate the Sholl morphology of individual microglia. The center of the soma was user defined, and intersections were calculated along the path of concentric circles starting 5 µm from the soma center and increasing by 1 µm in radius.

Volumetric interaction analysis

Tissue sections stained with multiple markers were imaged with Nikon AXR confocal microscope with either- (a) 40x water immersion objective (FOV: 435.35 x 435.35 μm^2) at 2048x2048 pixel resolution with 0.5 µm step size across a uniform 10 µm stack, or (b) 20x objective (FOV: 878.5 x 878.5 μm^2) at 2048x2048 pixel resolution with 1 µm step size across a uniform 10 µm stack. Setting used for a given experiment may be found in the results section. Volumetric interaction analysis was performed with imageJ using the following logic sequence: (1) Image volume was split into channels. (2) Stack thresholding was performed for each channel with 'stack histogram' enabled- (a) Otsu method for DAPI; (b) Li method for NeuN; (c) Triangle method for Iba1; (d) Triangle method for P2Y12; (e) Fixed threshold with bounds 25-255 (8-bit

images) for CD68. (3) CA3 ROI was drawn on z-projected DAPI volume (max-intensity method). (4) 'AND' function of image calculator was applied to a pair of thresholded channel volumes of interest to obtain a result stack that's double positive for both channels. (5) CA3 ROI was transferred to the result stack. (6) 'Analyze particles' function with no-size filter was applied to obtain a layer-by-layer area of the result stack within the CA3 ROI. (7) Area was multiplied by voxel thickness (0.5 µm for 40x images, 1 µm for 20x images) and summed to obtain the imaging volume positive for the chosen pair of channels of interest. (8) Double positive channel volume were then divided by the total CA3 volume (CA3 ROI area x total stack thickness of 10 µm) or Iba1 channel volume for normalization. Note: fixed bounds of threshold were used for CD68 due to poor performance of auto thresholding methods in sections with very low CD68. The bounds were empirically determined by visual inspection of parameter performance on the brightest and least bright sections of CD68 channel (max intensity z-projected images) among all the sections of all male animals examined.

Microglia soma size analysis

Soma size analysis was performed on Iba1 stained sections imaged with a Zeiss LSM 780 confocal microscope at 20x magnification (FOV: 424.26 x 424.26 μm^2), 1024x1024 pixel resolution with a 1 µm step size across a uniform 10 µm stack, centered on CA3. Using ImageJ, $Iba1^+$ $DAPI^+$ microglia nuclei within CA3 were found using the same logic sequence as described in microglia density analysis. Following additional steps were performed to obtain true soma- (1) Max-intensity z projected Iba1 images were auto thresholded using Otsu method. (2) Two iterations of 'open' function (process menu → binary → options) were applied to get rid of most of the microglia branches. (3) Four iterations of 'dilate' function (process → binary → dilate) were applied to the $Iba1^+$ $DAPI^+$ thresholded microglia nuclei to add material around them. (4) Image calculator with 'AND' function was applied to the results of steps (2) and (3) to obtain true soma, devoid of almost all branches. (5) Area and shape descriptors were enabled in

set measurements menu. (6) Analyze particles operation was performed with a size filter of 17 μm^2. (7) Circularity and area were noted to obtain the soma size and activation status respectively. ImageJ calculates circularity using the formula, circularity = 4 π (area/perimeter^2) where 1 indicates a perfect circle and values approaching 0 indicate increasingly elongated polygon.

In vitro microglia density analysis

Primary microglia separated from mixed culture were imaged at day 10 after replating with Keyence BZ-X800 fluorescence microscope at 20x magnification. Single z-planer images were obtained with TRITC (tdTomato) and phase contrast filters. ImageJ was used to quantify the number of tdT^+ cells in single fields of view per well by auto thresholding with Shanbag method and watershed (process → binary → watershed).

Assessment of culture purity

Culture purity was quantified as the percent of tdTomato positive cells among all the cells observed with phase contrast microscopy in the field of view (Fig.S3B).

Appendix 2: LC/MS analysis

Neuromodulators and amino acids were measured using liquid chromatography-mass spectrometry (LC-MS), as previously described (2, 3). Briefly, 20 uL sample was spiked with internal standards, deproteinized with a cold acetonitrile:methanol solution (50:50, v:v), and centrifuged at 18,000g for 15 minutes at 4°C. The supernatant was dried down and then immediately derivatized with 6-aminoquinolyl-N-hydroxysuccinimidyl carbamate, following the Waters AccQ-Fluor kit protocol. An 11-point calibration standard curve underwent a similar derivatization procedure after the addition of internal standards. Both the derivatized standards and samples were analyzed on a triple quadrupole mass spectrometer (Thermo TSQ Quantiva), coupled with an ultra-pressure liquid chromatography system (Waters Acquity UPLC). Data acquisition was performed using selective ion monitoring (SRM). Concentration of each analyte in the unknown sample was calculated using its respective calibration curve.

For in vitro metabolite release assay, measurements were also made in at least two blank isotonic and hypotonic solutions used in the experiment (negative controls). Similarly for in vivo experiment, blank isotonic PBS that was used to dilute the CSF was used as negative control. Average values of respective negative controls were subtracted from sample values (all samples) followed by correction for dilution (CSF only) before statistical comparison.

Appendix 3: Colorimetric assay for ATP

Isotonic and 30% hypotonic stock solutions were prepared as above with the addition of 100 µM ARL 67156 trisodium salt. ARL salt is an ectonucleotidase inhibitor and reduces hydrolysis of ATP. The metabolomics assay was repeated with the ARL added buffers. Following manufacturer's instructions, ATP standards were prepared separately for isotonic and hypotonic buffers in a range of 10 pM to 10 nM. ATP assay mix was loaded into a 96 well plate and rested for 3 minutes to hydrolyze endogenous ATP. Samples were added to the ATP assay mix and pipetted a few times. Luminance was read immediately on a spectrophotometric plate reader. Sample ATP values were derived from the standard calibration curves. Experiment was performed in a single batch and raw values were reported without any batch normalization.

REFERENCES

1. Ferreira TA, Blackman AV, Oyrer J, Jayabal S, Chung AJ, Watt AJ, et al. Neuronal morphometry directly from bitmap images. *Nature Methods.* 2014;11(10).
2. Lanza IR, Zhang S, Ward LE, Karakelides H, Raftery D, and Nair KS. Quantitative metabolomics by H-NMR and LC-MS/MS confirms altered metabolic pathways in diabetes. *PLoS One.* 2010;5(5):e10538.
3. Hinton DJ, Vazquez MS, Geske JR, Hitschfeld MJ, Ho AMC, Karpyak VM, et al. Metabolomics biomarkers to predict acamprosate treatment response in alcohol-dependent subjects. *Sci Rep.* 2017;7(1):2496
